# Validation of non-invasive methods for the measurement of gonadal and inter-renal steroid hormones in a desert-adapted amphibian (*Scaphiopus couchii*)

**DOI:** 10.1093/conphys/coaf007

**Published:** 2025-02-11

**Authors:** Alexander T Baugh, Callie Cho, Alice Onyango-Opiyo, Sophie A Rodner, Senna Mieth, Daniel Oakes, Liam Halstead

**Affiliations:** Department of Biology, Swarthmore College, 500 College Avenue, Swarthmore, PA 19081, USA; Department of Biology, Swarthmore College, 500 College Avenue, Swarthmore, PA 19081, USA; Department of Biology, Swarthmore College, 500 College Avenue, Swarthmore, PA 19081, USA; Department of Biology, Swarthmore College, 500 College Avenue, Swarthmore, PA 19081, USA; Department of Biology, Swarthmore College, 500 College Avenue, Swarthmore, PA 19081, USA; Department of Biology, Swarthmore College, 500 College Avenue, Swarthmore, PA 19081, USA; Department of Biology, Swarthmore College, 500 College Avenue, Swarthmore, PA 19081, USA

**Keywords:** Corticosterone, estradiol, saliva, stress, testosterone, validation, waterborne hormones

## Abstract

For aquatic and semi-aquatic vertebrates like amphibians, it is possible to estimate excreted hormone levels using non-invasive methods such as waterborne and salivary sampling. These techniques allow monitoring of endocrine activity over varying, repeated and simultaneous integration periods while minimizing handling-related stress that can ‘contaminate’ hormone estimates, including estimates of baseline glucocorticoids. Here we have validated the extraction and quantification of three steroid hormones (corticosterone, CORT; 17-b estradiol, E_2_; testosterone, TST) in Couch’s spadefoots (*Scaphiopus couchii*)—a desert-adapted anuran of special interest for physiology, evolution and conservation—using non-invasive waterborne and minimally invasive salivary hormone methods. We combined extraction and enzyme immunoassay methods to conduct conventional technical validations of parallelism, recovery and time-course. Next, we carried out biological validations by testing the correlation between excreted and circulating concentrations and conducting pharmacological challenges. We found that all three hormones can be precisely estimated from 60-min water baths, exhibit robust parallelism, and have high recoveries. Further, we demonstrated that secretory responses to pharmacological challenges can be detected in waterborne CORT in male and female frogs; in TST and E_2_ in male frogs, but not consistently for TST or E_2_ in female frogs. Lastly, plasma hormone concentrations were consistently correlated with their waterborne complements for CORT (both sexes), as well as TST and E_2_ in males (but not females). Plasma CORT was also positively correlated with salivary CORT. Together, our findings suggest that sampling waterborne and salivary hormones offers a minimally invasive method that field endocrinologists and conservation physiologists can use to obtain biologically informative endocrine estimates from desert-adapted amphibians.

## Introduction

Emerging threats, including climate and land use changes, infectious diseases and pollutants have contributed to declines and extinctions in a variety of amphibians (reviewed in Luedtke *et al.*, 2023). The use of biomarkers, including stress and reproductive hormones, to monitor population health and identify new and ongoing threats is steadily gaining interest (reviewed in Narayan *et al.*, 2019). The use of minimally invasive procedures is of particular interest given the vulnerable nature of many target species. Further, these methods offer additional advantages to behavioural endocrinologists because conventional invasive sampling procedures can perturb the very endocrine activity under study. This can drive the use of experimental designs that, because of this invasiveness problem, require that the measurement of behaviour precedes the sampling of hormones (i.e. introduce order effects). This is particularly problematic for endocrine systems such as the hypothalamic–pituitary–adrenal/inter-renal (HPA/I) axis wherein secretory activity can react quickly to certain experimental manipulations such as handling (Romero and Reed, 2005). Further, this activation can then rapidly feedback on neuroendocrine expression (Rodriguez-Santiago *et al.*, 2024), including in ways that interact with state-dependent (Baugh *et al.*, 2013, 2017a, 2021; Gall *et al.*, 2019), context-dependent (Baugh *et al.*, 2017b; Smit *et al.*, 2024,), and bidirectional effects on behaviour (Cheng, 1992; Baugh, 2024). These complications could be ameliorated by sampling methods that avoid or minimize these experimental perturbations. Hence, sampling matrices that admit to non-invasive procedures is critically important not merely from an animal-use and conservation perspective, but also from an experimental design perspective.

Although many technologies now exist for the real-time, non-invasive monitoring of behaviour (e.g. automated tracking and analysis, animal-deployed accelerometers), we do not yet have the biotechnologies that would permit real-time, non-invasive endocrine activity measurement in tissue. For aquatic and semi-aquatic organisms, however, there is an opportunity to measure excreted hormones from their natural environment (water) that could be both non-destructive and synchronized with automated, non-invasive behavioural measurements. These excreted hormone quantification procedures have now been developed for several fish (reviewed in Scott and Ellis, 2007) and amphibian species (reviewed in Narayan *et al.*, 2019), including in salamanders (*Eurycea nana*; Gabor *et al.*, 2013a), ranids (Forsburg *et al.*, 2019), common midwife toad (*Alytes obstetricans*; Gabor *et al.*, 2013a), túngara frogs (*Physalaemus pustulosus*; Baugh *et al.*, 2018; Baugh *et al.*, 2021), eastern grey treefrogs (*Hyla versicolor*; Bastien *et al.*, 2018), cane toads (Narayan *et al.*, 2012a), and Fijian ground and tree frogs (Narayan *et al.*, 2012b). Among these studies, glucocorticoids (GCs) are the most commonly validated steroids (reviewed in Ruthsatz *et al.*, 2023; but see Baugh and Gray-Gaillard, 2021; Baugh *et al.*, 2018; Ringler *et al.*, 2024), likely reflecting the fact that these steroids are more abundant in excreted matrices, as well as the potential relevance of GCs for animal conservation and welfare. However, many important domains of behavioural ecology and conservation physiology are centred on breeding biology, including *ex situ* approaches that may require the sensitive monitoring of reproductive status (Cikanek *et al.*, 2014); therefore, it is important that we also develop and refine methods for the non-invasive measurement of gonadal hormones.

Waterborne steroids likely reflect the input of multiple sources, including urinary (reviewed in Narayan *et al.*, 2019), salivary (Hammond *et al.*, 2018) and dermal excretions (Santymire *et al.*, 2018, 2021; Scheun *et al.*, 2019). Investigating these different inputs could provide a more complete understanding of source contributions and therefore optimal approaches for characterizing integration timelines and circulating hormone concentrations. Relative to waterborne approaches, salivary sampling involves handling restraint. However, this disadvantage might be offset with improved accuracy and/or precision of hormone estimates, and shorter sampling durations.

In the present study we evaluated three steroid hormones (corticosterone, CORT; 17-b estradiol, E_2_; testosterone, TST) in plasma and water, as well as salivary CORT. Technical and biological validations were conducted to determine whether these sampling methods accurately capture representative hormone profiles in female and male adult Couch’s spadefoots (*Scaphiopus couchii*). We used adult *S. couchii* because spadefoots are emerging as a model system in behavioural evolution and development due to some remarkable phenotypic plasticity. Tadpoles exhibit rapid, inducible changes in morphology and developmental rates (Newman, 1989; reviewed in Ledón-Rettig and Pfennig, 2011)—and a substantial effort has been underway to understand the role of hormones regulating these plastic traits and their evolution (Harvey *et al.*, 1997; Hollar *et al.*, 2011), particularly in larval spadefoots (Denver, 1998; Gomez-Mestre *et al.*, 2013; Burraco and Gomez-Mestre, 2016; Kulkarni *et al.*, 2017; Ledón-Rettig *et al.*, 2023).

Couch’s spadefoots are relatively large anurans (~60 mm snout-vent length, SVL) that inhabit the Chihuahuan and Sonoran deserts of North America (Stebbins,1985). These frogs hibernate in arid soils to avoid desiccating conditions. Further, as explosive breeders, they only spend a small fraction of their adult life in aquatic environments during the monsoons (Seymour, 1973; Woodward, 1982; Sullivan and Sullivan, 1985). For these reasons, *S. couchii* likely have some unique endocrine adaptations, including rapid secretory transitions and an osmoregulatory physiology adapted for water conservation (Dimmitt and Ruibal, 1980). Such anti-desiccation adaptations could introduce challenges for excreted hormone sampling, compared to more aquatic species that have been the targets of most of the previous research on non-invasive methods (Baugh *et al.*, 2018; Narayan *et al.*, 2012b; but see Ledón-Rettig *et al.*, 2023). Lastly, desert-adapted species like Couch’s spadefoots are particularly susceptible to climate and land use changes. Urbanization and agriculture (irrigation) has been particularly impactful in eliminating populations of *S. couchii*—because of these impacts, they are listed as a Species of Special Concern in California (Jennings and Hayes, 1994) and Colorado (https://cpw.state.co.us/species/couchs-spadefoot).

## Materials and Methods

### Animals

We used mature adult male and female *S. couchii* (*n* = 70) that were acquired from a commercial vendor in 2021 (Backwater Reptiles, Meridian, ID USA), which were housed, and maintained at constant temperature (21°C) in a humidity and light-controlled (L:D 14:10) vivarium facility at Swarthmore College and fed an *ad libitum* diet of vitamin-supplemented crickets. Experiments were conducted between 2021 and 2024 between 0800 and 1800. Sample sizes varied among experiments (see below), and some individuals were used in multiple experiments after a minimum of 14 days (maximum >24 months) from their previous use (we did not observe any carryover effects or age-effects). After balancing for sex, animals were randomly assigned to the treatments. They were typically housed in same-sex groups of two but occasionally in groups of up to four in identical terraria (L × W × D, cm: 54 × 29 × 32) with lightly moistened sand. Under these conditions, spadefoots burrow under the sand and remain dormant with the occasional and brief surfacing to feed. For each experiment, we dug up frogs, and immediately performed the sample collection; following sampling, we measured body mass using a digital scale (to the nearest 0.001 g; Sartorius Entris, Göttingen, Germany) and SVL using analogue callipers (to the nearest 0.01 mm; Avinet, Portland, ME, USA). This study was approved by the Institutional Animal Care and Use Committee at Swarthmore College (protocol number 081021).

### Experimental approach

Our validation study was comprised of five components: (1) testing for parallelism for each of the three matrices between serial dilutions of pooled samples and the standard curves; (2) estimating recovery efficiencies (i.e. extraction loss) for the two matrices that involved extraction (water, plasma) by adding a known quantity of exogenous hormone to each matrix and measuring loss during extraction; (3) estimating the linear correlation of hormone concentrations between matrices, particularly between plasma and the two excreted matrices; (4) validating various sampling durations and timelines for the excreted matrices; (5) testing the effects of pharmacological challenges versus control treatments on circulating and excreted hormone concentrations.

### Hormone sample collection, extraction and quantification

#### Plasma hormones

For circulating hormones, we rapidly (<3 min) collected blood (~50 μl) via a non-lethal cardiac puncture method without anaesthesia using a 30-gauge insulin syringe (BD Micro-fine U-100, 0.3 ml) pre-rinsed with heparin. We have used this procedure without adverse health effects in multiple species (Baugh *et al.*, 2019; Baugh *et al.*, 2021) and Bastien *et al.* (2018) demonstrated that this method accurately captures plasma CORT concentrations without any elevation due to handling. Whole blood was stored at 4°C for <1 h and then centrifuged (7500 rpm for 8 min at 8°C (Denville 260D, Denville Scientific, Inc.)). The plasma fraction was then collected and stored at −20°C for up to 4 weeks. Plasma hormones were extracted using an established double diethyl-ether liquid extraction procedure, and subsequently reconstituted in the assay buffer from the commercial kit (see below; Baugh *et al.*, 2018; Baugh and Gray-Gaillard, 2021).

#### Waterborne hormones

We conducted water baths according to procedures we have used in other anurans (see Baugh *et al.*, 2018; Baugh and Gray-Gaillard, 2021). First, we prepared ‘frog water’ for waterborne hormone sampling by dissolving 1.2 g CaCl2, 1.38 MgSO4, 1.08 g KHCO3 and 0.038 g of a commercial trace element (R/O Right, Kent Marine, Franklin, WI) into 30 l of reverse osmosis-purified water. We quickly transferred individual frogs directly into 100 ml of frog water within a 500-ml beaker (Supplementary Fig. S1) topped with a weighted mesh lid. We let them bathe 60 min (under silence) for all experiments except for the water bath duration experiment (see below). Following the water bath, we filtered water samples (reverse osmosis (RO) water-saturated coffee filters) to remove particles such as sand and finally rinsed the filtrate with RO water to increase recovery. For parallelism and sample recovery, individual 100-ml water baths were pooled, homogenized and stored at −80°C until thawing for solid-phase extraction (see below). For all other experiments, individual samples were maintained separately.

Water samples were solid-phase extracted according to previously published methods (Gabor *et al.*, 2013a; Baugh *et al.*, 2018; Baugh and Gray-Gaillard, 2021). First, we fitted ports on vacuum manifolds with 50 mg silica gel cartridges (United Chemical Technologies, LLC, Bristol PA; Sep-Pak C18, Waters Corp., Milford, MA). Cartridges were primed with 4 ml of ACS-grade MeOH, followed by 4 ml of deionized (DI) water. Samples were passed slowly through the cartridges under 15 bar of vacuum pressure, washed with 4 ml RO water and eluted with 3 ml of HPLC-grade MeOH into borosilicate vials. Eluents were then dried in a centrifuge concentrator (Savant SPD1010 SpeedVac Concentrator, Thermo Fisher Scientific, Waltham, MA) at 60°C for 4 h and frozen in a dry state at −20°C until reconstitution (see below).

#### Salivary hormones

We modified the protocol described in Hammond *et al.* (2018) for sampling saliva to measure salivary CORT (sCORT). We collected saliva samples using Salivette swabs (SalivaBio Infant’s Swab, Salimetrics LLC). First, we cut each swab into 35-mm sections with one section used per frog. Handling time was kept to a minimum by having each frog individually housed in a small plastic container (150-mm diameter) with sand prior (4–6 h) to sampling. Quickly, the frog’s mouth was gently opened using a sterile stainless steel spatula and the sterile swab was held in each frog’s mouth for 10 min using sterile forceps. We used a 10-min sampling duration because a pilot study using briefer durations did not yield sufficient saliva volumes. The saturated swab was then placed in the top of the Salivette centrifuge tube (with a plastic filter below) and frozen at −20°C, which has been shown to precipitate inferring mucins (Toone *et al.*, 2013). Immediately prior to assaying using enzyme immunoassay (EIA), we removed the swab/centrifuge tube from the freezer, and centrifuged under refrigeration (4°C) for 10 min at 7000 rpm to collect liquid (Sorvall X Pro Series, Thermo-Scientific). We transferred a known volume of saliva (50 μl per sample in most cases) to 2-ml microcentrifuge tubes and treated with trichloroacetic acid (TCA; 10 μl TCA per 50 μl saliva), which has been shown to precipitate interfering proteins (Cascalheira *et al.*, 2008). We then vortexed samples for 10 s, incubated for 15 min at 20°C and then centrifuged for 8 min at 6000 rpm. We then collected a known volume of supernatant and diluted it in commercial assay buffer at a ratio of 1:3, which was demonstrated in our parallelism component to be an optimal ratio, and then immediately performed the ELISA.

#### Enzyme immunoassays

We used commercial EIA kits to measure concentrations of CORT (DetectX® kit, Arbor Assays, Ann Arbor, MI; Cat. No. k014), TST (Cat. No. K032) and estradiol (waterborne: K030; plasma: KB030). Samples and reagents were brought to room temperature, and the reconstituted samples were vortexed and plated. Samples, non-specific binding and total binding controls, standards, spiked/stripped samples, and frog water-only controls were assigned to duplicate wells. Assays were carried out according to kit instructions, with plates read on a Versamax reader at 450 nm using a four-parameter curve-fitting equation in Softmax Pro (Molecular Devices, Sunnyvale CA).

For CORT, the lower limit of detection (lowest value of an analyte reliably detected in a native sample) was 17.5 pg ml^−1^, sensitivity (lowest value of analyte in assay buffer that the assay can statistically differentiate from background) was 20.9 pg ml^−1^ and the cross-reactivity (ability of antibody to bind given antigen) is 100% for CORT, 18.9% for 1-dehydrocorticosterone and 12.3% for desoxycorticosterone. E_2_ had a lower limit of detection and sensitivity of 2.05 and 2.21 pg ml^−1^ for plasma and 26.5 and 39.6 pg ml^−1^ for waterborne samples, respectively. The cross-reactivity of the antiserum for the plasma estradiol kit is 100% for estradiol, 3.2% for estrone sulfate and 2.5% for estrone. The cross-reactivity of the antiserum for the water estradiol kit is 100% for estradiol and 0.73% for estrone. TST had a lower limit of detection and sensitivity of 9.92 and 30.6 pg ml^−1^, respectively. The cross-reactivity of the antiserum is 100% for TST, 56.8% for 5α-dihydrotestosterone and 0.27% for androstenedione.

### Corticosterone

#### Parallelism and assay reliability

To determine optimal dilution factors and test for parallelism for waterborne CORT (wbCORT), sCORT and plasma CORT concentrations, we measured a range of serially diluted pooled samples. Extracted pooled water samples were serially reconstituted in the kit’s assay buffer solution at six dilution factors (1:1 (= 200 μl assay buffer solution), 1:2, 1:4, 1:8, 1:16 and 1:32). This dilution curve was compared to the standard curve of nine standards. We followed a similar protocol for sCORT, generating serial reconstitution dilutions of 1:4, 1:8, 1:16, 1:32 and 1:64. Lastly, with a pooled sample of plasma, we performed dilutions of 1:10, 1:20, 1:40, 1:80 and 1:160. We plotted standard curves and these dilution series to visually and statistically (difference of slopes *t*-test) evaluate parallelism (Baugh *et al.*, 2018). To estimate the reliability of our extraction and EIA procedures, determined coefficients of variation (%CV) by using 2–4 replicate individual samples (for each matrix) plated within and across plates. Additionally, we extracted and measured CORT on two separate aliquots of plasma from 16 frogs in order to evaluate technical reliability by testing for the linear correlation between the two replicate runs, which should then reflect the cumulative error introduced through variation in extraction efficiency, liquid handling and ELISA well variation.

#### Recovery

We subsampled 50 ml from each water sample from each of 16 animals (*n* = 7 m, 9 f) to estimate recovery efficiency. These subsamples were first pooled, homogenized and then aliquoted into 16 replicates. These were then split into four treatments: unspiked/unspiked (UU), unstripped/spiked (US), stripped/unspiked (SU) and stripped/spiked (SS) (*n* = 4 per treatment). Each of the 16 aliquots was then subdivided into equal thirds to create replicates for CORT, E_2_ and TST recovery determination. CORT was then extracted from each of the assigned aliquot thirds through solid phase extraction.

Endogenous steroids were stripped from half of the samples (SS and SU treatment groups) using 0.1 g of dextran-coated activated charcoal. These samples were then vortexed and incubated at 37°C for 4 h. After incubation, samples were centrifuged (4°C at 3750 RCF for 20 min), and the supernatant was collected. This stripping procedure was repeated three times for each sample (Delehanty *et al.*, 2015; Baugh *et al.*, 2018). Each sample was then solid phase extracted, dried, frozen and reconstituted with 533 μl of assay buffer solution at the optimal dilution of 1:16, adjusted for the proportion of a 60-min water bath. Additionally, half of the samples were spiked (US and SS treatment groups). Standard 1 from the CORT kit was prepared using RO water at 10 000 pg ml^−1^. The water samples were then spiked using a 50-μl subsample of standard 1. Each sample was then vortexed, placed in a −20°C freezer, processed through SPE and dried. The dried samples were reconstituted in assay buffer solution at 1:16, providing a final estimated exogenous CORT concentration of 937.55 pg ml^−1^. Final measured concentrations among each of these four sample types were determined, which allowed us to estimate percent recovery following the procedure in Baugh *et al.* (2018).

#### Water bath duration

We performed a series of variable water bath durations in order to determine the optimal duration for water sampling. The goal was to ascertain the minimum duration required to obtain detectable CORT levels as well as to determine if there is an optimum duration—the shortest duration that minimizes inter-individual variance as the release rates stabilize. We used 20 frogs (10 m, 10 f) with four unique frogs (2 m, 2 f) subjected to one of five water bath durations (5, 10, 20, 40, 80 min).

#### ACTH challenges

‘ACTH Experiment 1’ examined wbCORT and ‘ACTH Experiment 2’ examined sCORT. Adrenocorticotropic Hormone (ACTH) is a reliable secretagogue of CORT via the hypothalamic–pituitary–inter-renal (HPI) axis that has been previously used to biologically validate non-invasive hormone measurements in amphibians (Narayan *et al.*, 2011; Gabor *et al.*, 2016; Baugh *et al.*, 2018; Forsburg *et al.*, 2019). In ‘ACTH Challenge Experiment 1’, animals were randomly assigned to receive either an intraperitoneal, mass-specific injection of ACTH (1 μg g^−1^ of Sigma A6303 porcine pituitary ACTH administered in dosages/volumes of 100, 150, or 200 μl according to bodyweight; *n* = 6), an injection of phosphate buffered saline (PBS) only (*n* = 6) or no injection (*n* = 5). Baseline wbCORT was measured 24 h prior to treatment (WB1), then again 30 min (WB2) and 24 h (WB3) after treatment. Blood was sampled immediately following WB2 in order to evaluate the correlation between wbCORT and plasma CORT.

In ‘ACTH Challenge Experiment 2’, we measured CORT in saliva samples 1 h prior to and at two time points following (0:45 and 2:45; h:mm) injection with either ACTH (0.1 μg g^−1^; *n* = 7, 3 m/4 f) or vehicle (PBS; *n* = 6; 3 m/3 f). Following the final saliva sample, we rapidly collected a whole blood sample via cardiac puncture, which allowed us to also estimate the correlation between circulating and sCORT. We analysed the effects of these experimental treatments using repeated measures general linear models (SPSS v.21; IBM).

#### Inter-matrix correlations

We measured the correlation between plasma and wbCORT, plasma and sCORT as well as wbCORT and sCORT. First, we collected a single baseline water bath for wbCORT followed immediately by blood sampling for plasma CORT using 20 unmanipulated frogs (10 m, 10 f). Next, using the frogs in ‘ACTH Challenge Experiment 1’, we also evaluated this waterborne and plasma CORT correlation. Lastly, from ‘ACTH Challenge Experiment 2’, we estimated the correlations between plasma and sCORT as well as between waterborne and sCORT. We used linear regression analysis to test these correlations.

### Testosterone and estradiol

#### Parallelism and assay reliability

As with CORT, we collected and pooled individual water baths from six males and separately for six females. Blood was collected, pooled and plasma was likewise serially diluted for parallelism. We determined whether serial dilution curves of waterborne TST (wbTST) and E_2_ (wbE_2_) (1:1, 1:2, 1:4, 1:8, 1:16, 1:32) as well as plasma TST and E_2_ (1:10, 1:20, 1:40, 1:80) were parallel to their standard curves. This also allowed us to determine the optimal dilutions for these steroids and matrices. Likewise, we used replicate individual unknown samples for both matrices determine intra- and inter-assay CVs.

#### Recovery

We replicated the basic procedures for recovery estimation described above for CORT in both TST and E_2_ in water and plasma following Baugh *et al.* (2018). We used 16 frogs (8 m, 8 f) from which we collected and pooled (within sex) water samples that were divided into the same four stripping/spiking treatments as described for CORT. We spiked water samples with the TST concentrate (from kit) to a target concentration that theoretically should occur within the linear portion of the standard curve (1204 pg ml^−1^). Likewise, samples were spiked with the kit’s E_2_ to a concentration of 19.8 pg ml^−1^. Samples, blanks and standards were plated in duplicate, except for the E_2_ samples, which were plated singly.

**Figure 1:**
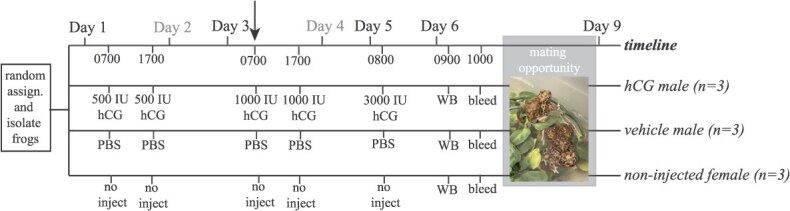
Timeline for the hCG challenge experiments. Six male frogs were injected on Day 1, 3 and 5 (three frogs with the indicated dosages (International Units, IU) of hCG diluted in PBS; and three frogs with the PBS vehicle) and three females were unmanipulated. For all nine frogs, we sampled waterborne estradiol and TST with a single 60-min water bath (WB) followed immediately by blood sampling for plasma estradiol and TST and then a 72-h window of co-housing of all nine frogs with water.

#### GnRH challenge experiment

Injections of gonadotropin-releasing hormone (GnRH) have been used to successfully stimulate breeding in species closely related to *S. couchii* (*Spea multiplicata & Spea bombifrons*; Seidl *et al.*, 2019). Therefore, we performed an experiment using a GnRH dose used in Seidl *et al.* (2019) alongside a vehicle treatment (PBS), while sampling waterborne and plasma TST and E_2_, at two pre-injection and four post-injection time points, including one time point (WB5) occurring 24 h following injection, which is the predicted peak gonadal response to GnRH based on previous studies (Baugh and Gray-Gaillard, 2021; Supplementary Fig. S2). We used 20 frogs (*n* = 10 f, *n* = 10 m) randomly assigned but balanced to receive either an injection of GnRH (0.7 μg GnRH/frog in 70 μl PBS; Sigma Cat. No. 35263–73-1) or an injection of the vehicle control (70 μl PBS). Waterborne hormone samples were taken 48 and 24 h prior to intraperitoneal injections, which were performed with 31 gauge, 1-ml needles (BD Designs). Waterborne hormone samples were again taken 6, 12 and 48 h after injections, with the 6-h sample being immediately followed by a blood sample for plasma-wb correlation testing. We performed separate repeated-measures general linear models (GLMs) for wbTST and wbE_2_. Both models incorporated the between-subjects factors of sex and treatment with the six wbTST (or wbE_2_) samples as the sole response variable. Lastly, using this dataset we also tested for the linear correlations between plasma TST and wbTST (and plasma E_2_ vs wbE_2_).

#### hCG challenge experiment

Given the inconsistent results in the GnRH Challenge Experiment, we conducted pharmacological challenges (Fig. 1) using an alternative secretagogue–human chorionic gonadotropin (hCG; Sigma Cat. No. 9002-61-3), which we have used successfully in other frogs to stimulate gonadal hormone secretion and excretion (Baugh *et al.*, 2018) and sexual behaviour readiness (Baugh and Ryan, 2010; Baugh *et al.*, 2012). We injected (i.p.) three males with a series of increasing dosages of hCG (or vehicle, PBS) using 31 gauge, 1-ml needles (BD Designs) over the course of several days alongside three unmanipulated females (see Fig. 1). We elected to not challenge females given the results of the GnRH experiment, which showed a male but no female responsiveness to pharmacological challenge. Twenty-four hours following the final treatment we sampled for wbTST and wbE_2_, followed by blood sampling. Lastly, we then co-housed all 9 experimental frogs in a large open aquarium facility with artificial surface plants and monitored them for breeding.

#### Inter-matrix correlations

Using the water and plasma samples collected from the frogs used in the *GnRH* and ‘hCG Challenge Experiments’, we performed linear regression analyses to determine if wbTST and wbE_2_ concentrations were correlated with circulating levels.

### Statistics

All hormone concentrations were log_10_ transformed to improve residuals prior to analysis. Following transformation, assumptions of sphericity, homogeneity of variance and Gaussian error distributions were met. Where multiple comparisons were made, we have expressed *P*-values using the Sidak correction.

## Results and Discussion

Overall, our results demonstrate that waterborne hormone sampling is an accurate and biologically informative method for estimating CORT and TST and moderately effective for E_2_ in *S. couchii*. In both sexes, CORT is rapidly excreted in water and saliva in abundant concentrations, stabilizes in the rate of excretion at 40-min (or longer) durations, responds robustly and quickly to ACTH challenge and is reliably correlated with plasma CORT concentrations. Similarly, TST is abundant in water, exhibits a strong sex difference (high TST in males), responds in a sex-specific manner to pharmacological challenge and is strongly correlated with plasma TST concentrations. Likewise, salivary sampling captures accurate and biologically informative estimates of CORT.

### Corticosterone

#### Parallelism and assay reliability

We found robust parallelism for CORT in all three matrices and found that the optimal dilutions for CORT (linear portion of the standard curve) were 1:50 for plasma (Supplementary Fig. S3), 1:16 (= 3.2 ml reconstitution buffer @ 1:1 = 200 μl buffer) for water (Supplementary Fig. S4) and 1:3 for saliva (Supplementary Fig. S5). In all cases, the serial dilution curves were not significantly different in slope to the standard curves (see Supplementary Material). These parallelism results resemble what we have reported in other frog species using similar methods (Bastien *et al.*, 2018; Baugh *et al.*, 2018). Coefficients of variation (%CV) were calculated using 2–4 replicate individual samples (for each matrix) plated within and across plates, and were within the typical range for plasma (intra-assay = 1.5%; inter-assay = 0.8%), water (intra-assay = 7.5%; inter-assay = 0.8%) and saliva (intra-assay = 6.9%; interassay = 11.9%). Lastly, we extracted and measured CORT on two separate aliquots of plasma from 16 frogs in order to evaluate technical reliability—the cumulative error introduced through variation in extraction efficiency, liquid handling and ELISA well variation. The correlation between concentrations measured on the first and the second aliquot (within 1 plate) indicated strong consistency (r = 0.98, *n* = 16, *P* < 0.00001). Control samples (pure ‘frog water’ in which no frog was held) had undetectable levels of wbCORT.

#### Recovery

We recovered an average of 81.5% (±1.3% SEM) of CORT from 60-min water baths. This represents a moderately high and invariant (across samples) recovery efficiency, indicating low technical error during extraction and EIA and suggesting that these methods are sufficient, and individual recoveries (e.g. using RIA) are not necessary. Further, these recovery estimates are similar to what we have reported in other frog species using similar methods (Bastien *et al.*, 2018; Baugh *et al.*, 2018). Because saliva samples did not involve an extraction step, recoveries were not estimated.

**Figure 2:**
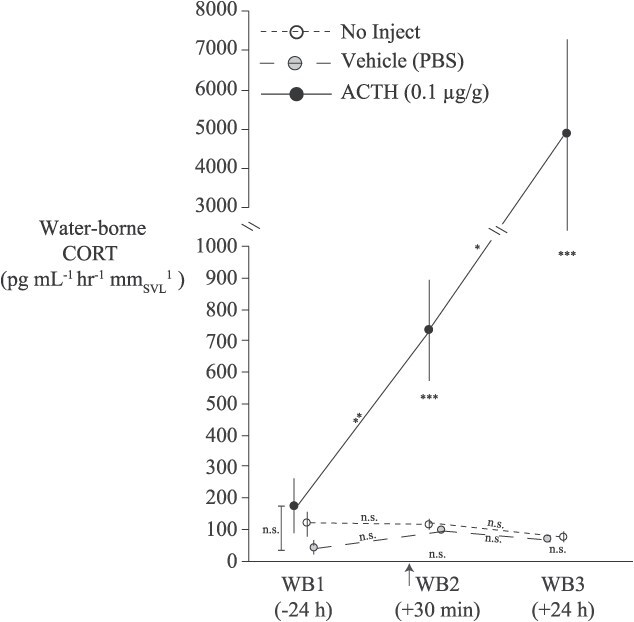
Waterborne CORT concentrations (mean ± SE) before and at two time points after injection with either vehicle (PBS; *n* = 5), ACTH (0.1 μg g^−1^; *n* = 7), or no injection (*n* = 6). Note that the y-axis is interrupted with a scale change in order to represent WB3 concentration for the ACTH group. Intercepts along the abcissa are jiggered for visualization. Symbols of statistical significance illustrated along the lines connecting time points within a treatment group indicate the pairwise *post hoc* tests comparing the two sequential time points within each treatment (^*^P < 0.05, ^**^P < 0.01; ^***^P < 0.001; Sidak-corrected for multiple comparisons). The remaining symbols indicate the pairwise *post hoc* tests comparing treatment groups within each time point. The arrow on the x-axis indicates the timing of the injection/handling (30 min prior to WB2). Statistical analyses were performed on log_10_-transformed values though untransformed values are depicted here to aid in interpretation.

#### Water bath duration

A 5-min water bath was sufficient to measure wbCORT, and levels increased (presumably through accumulation) over longer durations (Supplementary Fig. S6). Expressing the wbCORT levels per minute of water bath duration yielded CORT release rates, which indicated that durations <40 min exhibit considerably more variation among frogs in wbCORT; in contrast, water bath durations >40 min exhibit consistent release rates. For this reason, we elected to proceed with 1-h water baths throughout this study, which is consistent with other studies (Gabor *et al.*, 2013a; Bastien *et al.*, 2018; Baugh *et al.*, 2018). If needed, future investigators could conceivably use shorter sample collection durations.

#### Pharmacological challenges

In ‘ACTH Challenge Experiment 1’ we found that the ACTH injection rapidly elevated wbCORT, whereas both control treatments did not experience changes in excreted CORT levels (Fig. 2). In other words, the waterborne sampling and injection *per se* do not appear to evoke an endocrine stress response in this species, which is consistent with what we previously reported in a tropical frog (Baugh *et al.*, 2018). In contrast, Gabor *et al.* (2016) showed that San Marcos salamanders (*E. nana*) respond with a CORT increase following injection. We conducted a GLM with a repeated statement to analyse these data that included an interaction term between treatment and time point. This interaction was significant (F_4,26_ = 4.8, *P* = 0.005) indicating that CORT changed differently among the three treatments. Pairwise comparisons of the treatment groups indicated that the control groups did not differ from each other (*P* = 0.89), whereas the ACTH group differed from both the no-inject (*P* = 0.0001) and vehicle (*P* < 0.0001) control groups. There were no differences between any of the treatment groups at the pre-injection (WB1) time point (all *P* > 0.14). Further, the two control groups did not differ from each other at the two post-injection time points (both *P* > 0.96), whereas both control groups differed from the ACTH treatment at both post-injection time points (both *P* < 0.0001). Lastly, in the ACTH treatment group, there was a significant increase in waterborne CORT between WB1 and WB2 (*P* = 0.01) and again between WB2 and WB3 (*P* = 0.04).

**Figure 3:**
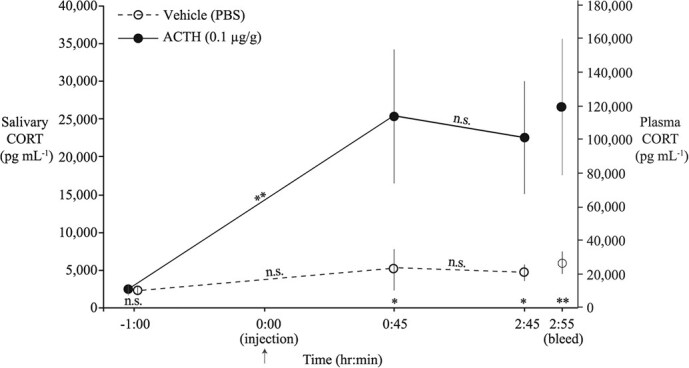
Salivary CORT concentrations (mean ± SE) before and at two time points after injection (arrow) with either vehicle (PBS; *n* = 6) or ACTH (0.1 μg g^−1^; *n* = 7). Symbols for statistical significance illustrated along the x-axis indicate the pairwise *post hoc* tests comparing the two treatment groups within each time point, whereas the symbols illustrated along the lines connecting points indicate the pairwise *post hoc* tests comparing the two sequential time points within each treatment (^*^P < 0.05, ^**^P < 0.01; Sidak-corrected for multiple comparisons). The right y-axis represents the plasma CORT concentrations (mean ± SE) sampled immediately following the final saliva sample (at 2:55 on the x-axis). Statistical analyses were performed on log_10_-transformed values but untransformed values are depicted here to aid in interpretation.

In ‘ACTH Challenge Experiment 2’ we found that sCORT levels increased following injection with ACTH but not following vehicle injection (treatment*time point; F_2,20_ = 5.82, *P* = 0.01; Fig. 3). There was no difference in sCORT levels between the ACTH and vehicle treatment groups at the pre-injection time point (−1 h; *P* = 0.54). The sCORT levels became highly elevated in response to ACTH injection at both post-injection time points relative to their pre-injection levels (Fig. 3). Lastly, plasma CORT concentrations were significantly higher in ACTH-injected frogs compared to the vehicle treatment (t_11_ = 3.51; *P* = 0.005; Fig. 3).

#### Inter-matrix correlations

**Figure 4:**
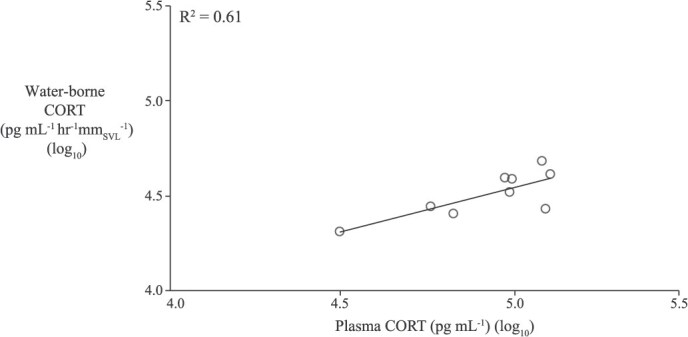
The linear correlation between plasma and waterborne CORT (*n* = 9; log_10_-transformed) from ACTH (0.1 μg g^−1^)-injected frogs. Blood was sampled immediately following the water bath.

Circulating concentrations of CORT were positively correlated with wbCORT in unmanipulated frogs (R^2^ = 0.24; F_1,18_ = 2.8; *P* = 0.028; *n* = 20; Supplementary Fig. S7) and likewise in a separate sample of ACTH (0.1 μg g^−1^) injected frogs (R^2^ = 0.61; F_1,7_ = 10.8; *P* = 0.013; *n* = 9; Fig. 4).

**Figure 5:**
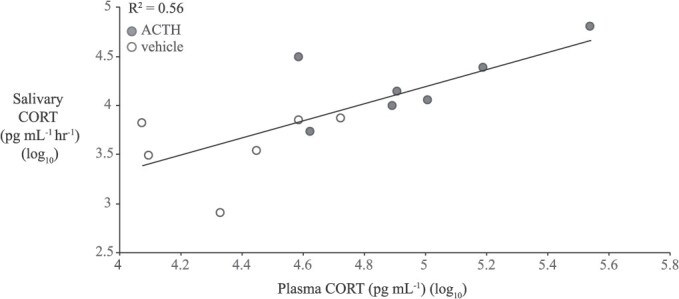
The linear correlation between plasma and salivary CORT (*n* = 13; log_10_-transformed). Blood was sampled immediately following the final saliva sampling (+2:45, h:mm). Frogs from both treatment groups (ACTH and saline-injected) are included in the correlation.

The sCORT (from ‘ACTH Challenge Experiment 2’) sampled immediately before bleeding (i.e. the 2:45 time point) was positively correlated with plasma CORT (R^2^ = 0.56, F_1,11_ = 14.2, *P* = 0.003; *n* = 13; Fig. 5); the plasma CORT versus sCORT correlation was weaker and non-significant at the +45-min saliva time point (R^2^ = 0.29, F_1,10_ = 4.13, *P* = 0.07; *n* = 12), which incorporated a larger interval between saliva and blood sampling (2 h). Lastly, sCORT was positively correlated with wbCORT when the two matrices were sampled in immediate succession (R^2^ = 0.38; F_1,11_ = 6.74, *P* = 0.025; Supplementary Fig. S8).

### Testosterone and estradiol

#### Parallelism

We established parallelism for TST in both plasma and 60-min water baths and determined that the optimal dilutions for TST were 1:10 for plasma (Supplementary Fig. S9), 1:8 (= 1.6 ml reconstitution @ 1:1 = 200 μl buffer) for water in males (Supplementary Fig. S10) and 1:3 for water (= 600 μl reconstitution) in females (Supplementary Fig. S11). In all cases, the serial dilution curves were not significantly different in slope to the standard curves (see Supplementary Materials). Coefficients of variation (%CV) were calculated using 2–4 replicate individual samples (for each matrix) plated within and across plates, and were within the typical range for plasma (intra-assay = 10.7%) and water (intra-assay = 2.2%; inter-assay = 4.7%).

Similarly, we established parallelism for E_2_ in both plasma and water and estimated that the optimal dilutions for E_2_ were 1:5 for plasma (Supplementary Fig. S12), 1:4 (= 800 μl reconstitution @ 1:1 = 200 μl buffer) for water in males (Supplementary Fig. S13) and 1:2 for water (= 400 μl reconstitution) in females (Supplementary Fig. S14). In all cases, the serial dilution curves were not significantly different in slope to the standard curves (see Supplementary Materials). Coefficients of variation (%CV) were calculated using 2–4 replicate individual samples (for each matrix) plated within and across plates, and were within the typical range for plasma (intra-assay = 5.2%) and water (intra-assay = 2.6%; inter-assay = 4.5%). Control samples (pure ‘frog water’ in which no frog was placed) had undetectable levels of wbTST and wbE_2_.

#### Recovery

We recovered an average of 83.1% (±6.9% SEM) of wbT. This represents a moderately high and consistent recovery efficiency, indicating low technical error during extraction and EIA, and suggesting that these methods are sufficient and individual recoveries (e.g. using RIA) are not necessary. Further, these recovery estimates are higher than what we have reported in another frog species using similar methods (Baugh and Gray-Gaillard, 2021).

**Figure 6:**
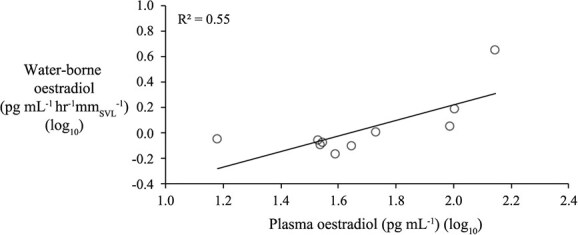
Circulating concentrations of estradiol were significantly correlated with waterborne estradiol in male frogs from the GnRH Challenge experiment at the second water bath time point.

We recovered an average of 86.3% (±9.5% SEM) of wbE_2_. This represents a moderately high and consistent recovery efficiency, indicating low technical error during extraction and EIA, and suggesting that these methods are sufficient and individual recoveries (e.g. using RIA) are not necessary. Further, these recovery estimates are similar to what we have reported in other frog species using similar methods (Baugh *et al.*, 2018). These technical validation results resemble what have been reported in other frog species using similar methods (Baugh *et al.*, 2018; Baugh and Gray-Gaillard, 2021).

#### GnRH challenge experiment

Injections of GnRH significantly elevated wbTST in males at 24 h following injection, but no consistent effects of treatment were detected in females (Supplementary Fig. S2). The GLM demonstrated that wbTST varied significantly across the six time points (F_5,12_ = 4.4, *P* = 0.017) but there were no significant main effects of treatment (F_1,16_ = 0.1, *P* = 0.76) or sex (F_1,16_ = 0.53, *P* = 0.47), but there was a significant treatment^*^sex interaction (F_1,16_ = 5.4, *P* = 0.033), which was due to the fact that males injected with GnRH had high wbTST, whereas females injected with PBS had high wbT. The fifth water bath (+24 h after treatment, i.e. the predicted peak gonadal activity; Baugh and Gray-Gaillard, 2021) was the only one in which the two treatments differed significantly (*P* = 0.001, *post hoc* pairwise comparison), which is driven by a robustly elevated wbTST in the GnRH-treated males at this predicted peak time point compared to WB4 (*P* = 0.016; Sidak-corrected *post hoc* pairwise comparison; Supplementary Fig. S2).

Similar to the patterns observed in wbT, injections of GnRH significantly elevated wbE_2_ in males at 24 h following injection, but no consistent effects of treatment were detected in females. The GLM demonstrated that wbE_2_ varied significantly across the six time points (F_5,12_ = 8.2, *P* = 0.001) but there were no significant main effects of treatment (F_1,16_ = 0.003, *P* = 0.95) or sex (F_1,16_ = 0.24, *P* = 0.63), but there was a significant treatment^*^sex interaction (F_1,16_ = 5.4, *P* = 0.05), which was due to the fact that males injected with GnRH had high wbE_2_. None of the six water bath time points differed significantly between the two treatments (all *P* > 0.05); however, similar to what we observed in wbT, wbE_2_ was selectively elevated in the GnRH-treated males at WB5 compared to WB4 (*P* = 0.048; Sidak-corrected *post hoc* pairwise comparison; Supplementary Fig. S2).

#### hCG challenge experiment

Waterborne and plasma TST were elevated in the hCG-injected males compared to vehicle-injected males and non-injected females (Supplementary Fig. S15). Similarly, wbE_2_ was somewhat higher in the hCG-injected males compared to vehicle-injected males and non-injected females; females, however, had the highest plasma E_2_ (Supplementary Fig. S15). One hCG-injected male had substantially higher wbE_2_ and this sample likewise had highly elevated waterborne and plasma TST (Supplementary Fig. S15). The following day, only the males injected with hCG were found in amplexus with the females and remained in amplexus for the duration of the 3-day observation period (Fig. 1).

#### Inter-matrix correlations

The correlation between plasma TST and wbTST in the frogs (both sexes combined) from the GnRH Challenge Experiment (at time point 5—the predicted peak that followed 24 h after the GnRH injection), though positive, was non-significant (R^2^ = 0.17; F_1,7_=; *P* < 0.07; *n* = 20; Supplementary Fig. S16). However, separating males and females in this dataset yielded some strong plasma–water correlations in males, but not females. Plasma TST was positively correlated with wbTST in males from the second (r = 0.91, *P* = 0.003), fourth (r = 0.73, *P* = 0.016) and sixth (r = 0.65, *P* = 0.041) water bath time points (Supplementary Fig. S17). Likewise, plasma E_2_ was positively correlated with wbE_2_ in males at the second water bath time point (r = 74, *P* = 0.014; Fig. 6). Moreover, plasma TST was positively correlated with plasma E_2_ in males (r = 0.77, *P* = 0.01). In females, plasma E_2_ and plasma TST were not significantly correlated with their waterborne complements at any time point (all *P* > 0.05). Lastly, at each of the six water bath time points in the ‘GnRH Challenge Experiment’, wbTST was positively correlated with wbE_2_ (R^2^ = 0.43–0.85; all *P* < 0.002; *n* = 20; both sexes; Supplementary Fig. S18).

**Figure 7:**
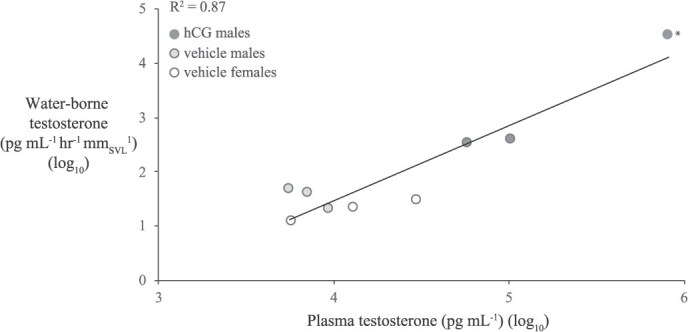
The linear correlation between plasma and waterborne TST (*n* = 9; log_10_-transformed) from frogs (all three treatment groups) in the hCG experiment. Blood was sampled immediately following the water bath. Removal of the datum in the far upper-right quadrant (*), does not qualitatively change the positive correlation.

Circulating concentrations of TST were positively correlated with wbTST in the frogs (both sexes) from the hCG Challenge Experiment (R^2^ = 0.87; F_1,7_ = 46.4; *P* < 0.001; *n* = 9; Fig. 7); when a datum with high leverage was removed, the positive correlation was retained (R^2^ = 0.67; F_1,6_ = 12.4; *P* = 0.01; *n* = 8; Fig. 7). Together, the GnRH and hCG Challenge Experiments demonstrated significant circulating–excreted correlations within males, and especially for TST in males. Females show weak or absent correlations between circulating and excreted gonadal hormones, perhaps indicating a sexually refractory state. This latter conjecture is consistent with the lack of oviposition observed at the end of the hCG Challenge Experiment, despite sexual readiness behaviour (clasping) observed in the males (Fig. 1).

#### Conclusions

We demonstrated that excreted gonadal and inter-renal hormones in adult spadefoots are relatively easy to sample and to accurately measure, reflect biologically informative estimates of individual (co)variation in circulating concentrations as well as the effects of endocrine stimulation. CORT and TST are abundant in excreted matrices in both males and females, which reflects the higher concentrations in circulation. Although excreted E_2_ is easily quantifiable, it’s lower absolute concentrations might make it a less reliable proxy, especially in non-breeding animals (e.g. females in the current study). Alternatively, excreted E_2_ might serve as a weak proxy for circulating levels due to potential differences in how oestrogens are metabolized and cleared. Further, we demonstrated that a 1-h water bath is sufficient to minimize error variance in excretion rates, though shorter durations are also practical.

In the current study sCORT yielded strong correlations with plasma (R^2^ = 0.56), considerably higher than what has been reported in the only other validation study (using a wild ranid) with circulating CORT measurements (R^2^ = 0.21; Tornabene *et al.*, 2023). In the present study in *S. couchii*, sCORT appears equivalent in quality with wbCORT—the correlations with plasma levels were similarly high as is the effort and costs required to conduct sample preparation. Saliva sampling has the benefit of a reduced sampling duration (10 min instead of 60 min) but is also considered more invasive than water sampling (Tornabene *et al.*, 2023). The poikilothermic physiology of anuran amphibians might explain the typically delayed GC response to stressors (Narayan *et al.*, 2013). Nevertheless, it is possible that the 10-min saliva sampling (and restraint) used in the present study induced the beginning of an acute stress response, thereby ‘contaminating’ what would otherwise be a baseline CORT estimate. While the water sampling method did not itself induce a wbCORT increase, the more intensive handling involved in salivary sampling might have. Hammond *et al.* (2018) demonstrated in three ranids that a salivary sample collected 15 min following handling had elevated sCORT concentrations. Similarly, Tornabene *et al.* (2023) showed that repeated handling in Columbia spotted frogs (*Rana luteiventris*) causes enduring elevations in sCORT that can last for days. Future refinement of this saliva sampling to reduce either its duration (e.g. 3 min) or the restraint intensity is needed. Alternatively, further development of dermal sampling is important (Santymire *et al.*, 2018, 2021; Scheun *et al.*, 2019) and could offer a rapid and minimally invasive sampling procedure.

To our knowledge, this is the first validation study of non-invasive methods in a desert-dwelling anuran. Our results are generally aligned with those found in other amphibians (Narayan *et al.*, 2019), suggesting that these methods are effective not merely with aquatic anurans but also for species with potentially divergent osmoregulatory regimes adapted for arid climates (McClanahan, 1964). While evolutionary and behavioural research has been conducted in this group of frogs (Woodward, 1982; Kelly *et al.*, 2021), there are also many unanswered questions about their physiology. For example, these non-invasive methods could be used to understand the mechanisms of rapid and environmentally inducible (e.g. pond drying) adaptive phenotypic plasticity observed during tadpole development (Newman, 1989; Crespi and Denver, 2004; Gomez-Mestre and Buchholz, 2006; Hollar *et al.*, 2011; Gabor *et al.*, 2013b; Kulkarni *et al.*, 2017). Similarly, what is the hormonal basis for how environmental cues like rainfall and groundborne vibrations stimulate emergence and rapid reproductive readiness in hibernating adults (Ruibal *et al.*, 1969; Seymour, 1973; Harvey *et al.*, 1997). Additionally, we think adapting these methods for use in the field to study how behaviour and endocrine activity are linked in anurans holds promise. For example, given the ability to rapidly and precisely sample GCs and androgens, it could be possible to directly measure male sexual behaviour (e.g. vocalization; Leary and Baugh, 2020) while simultaneously monitoring these key steroids repeatedly (e.g. via a flow-through system) over the relevant timeframes that appear to underlie (Still *et al.*, 2019) and potentially interact synergistically with behavioural output, including mating itself (Orchinik *et al.*, 1988; Baugh, 2024).

Together, methods like the ones validated here open up opportunities to address questions in conservation physiology for threatened or sensitive amphibians. This is especially applicable for small species, where tissue sampling (e.g. cardiac puncture) methods are impractical, highly invasive (Rexer-Huber *et al.*, 2012) or even lethal (Owen *et al.*, 2018). One important avenue in need of further investigation for excreted hormone and conservation in adult amphibians is improved specificity of the analyte. Water samples may contain both conjugated (from urine or faeces) and unconjugated (waterborne) steroids, whereas unconjugated or free steroids are the physiologically active form (McClelland and Woodley, 2021). Most studies to date lack the specificity of antibodies or extraction methods to distinguish these components as well as other steroid metabolites that might have considerable cross-reactivity. It is clear, however, that excreted steroid profiles are different from circulating profiles; e.g. in adult tungara frogs, cortisol is found (along with other GC metabolites) in water samples at relatively high levels but virtually absent in plasma (Baugh *et al.*, 2018). Future efforts to establish more comprehensive metabolite panels would be useful, especially since these approaches can simultaneously address additional questions related to biomarkers of physiological status and stress (Nagel *et al.*, 2019).

## Supplementary Material

Web_Material_coaf007

## Data Availability

Data will be made available on request.
