# Supplementary material for: Validation of non-invasive methods for the measurement of gonadal and inter-renal steroid hormones in a desert-adapted amphibian (Scaphiopus couchii)
Source: Conserv Physiol. 2025 Feb 11;13(1):coaf007. doi: 10.1093/conphys/coaf007 (PMC11821354; doi:10.1093/conphys/coaf007)
Supplement: Web_Material_coaf007 [file web_material_coaf007.zip › all_newSM1-18.pdf]

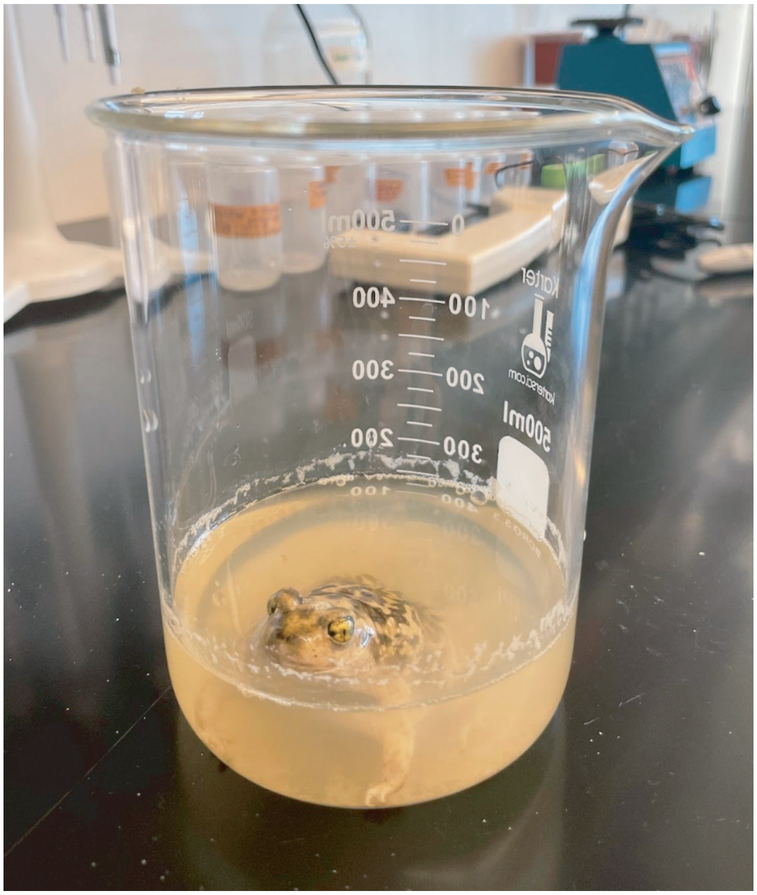

SM1. Photograph of an adult S. couchii in a 500 mL beaker with 100 mL of water (mesh lid not depicted). Frogs were continuously immersed during the sampling period.

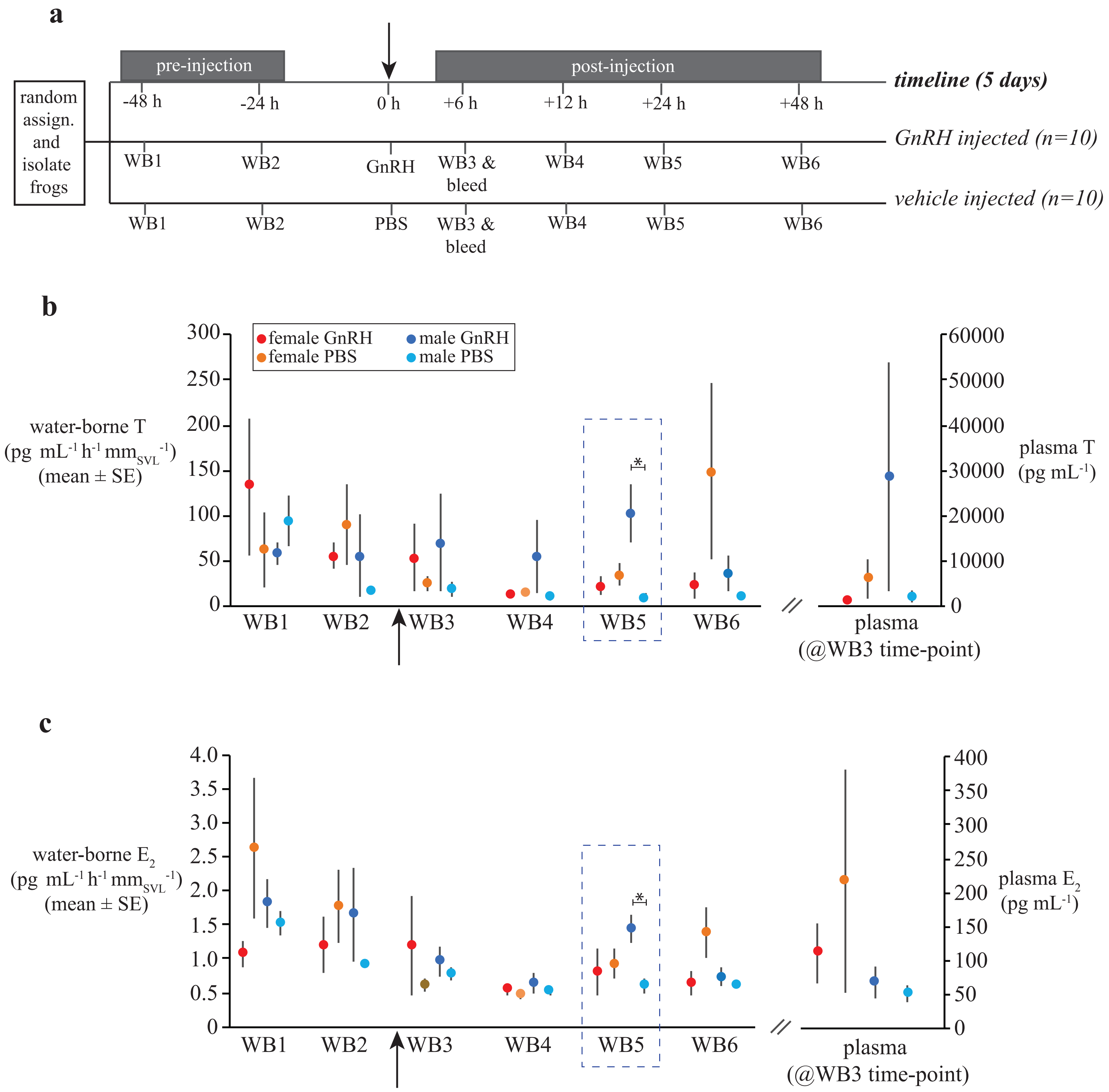

SM2. GnRH challenge experimental design and results for testosterone and estradiol.
(a) Timeline for the GnRH challenge pilot experiment. In June 2022, 5 male and 5 female frogs were randomly assigned to either the experimental (0.03 µg g^{-1} of GnRH) treatment or control (vehicle, PBS). Water bath samples (60 min) were collected at two time-points (WB1, WB2) before injection (arrow) and four time-points after injection (WB3-6). A blood sample (via cardiac puncture) was collected immediately following WB3 (6 h after injection). Testosterone (b) and estradiol (c) concentrations in water and plasma at the six WB timepoints. The arrows indicate the injection time and the dashed box highlights the 24 h post-injection water bath when peak gonadal hormones were predicted in water; male testosterone and estradiol was observed to be considerably elevated at this timepoint, and similarly elevated levels of T in GnRH males were observed in plasma, whereas females exhibit elevated plasma estradiol. *$p<0.05$, Sidak corrected planned pairwise comparison.

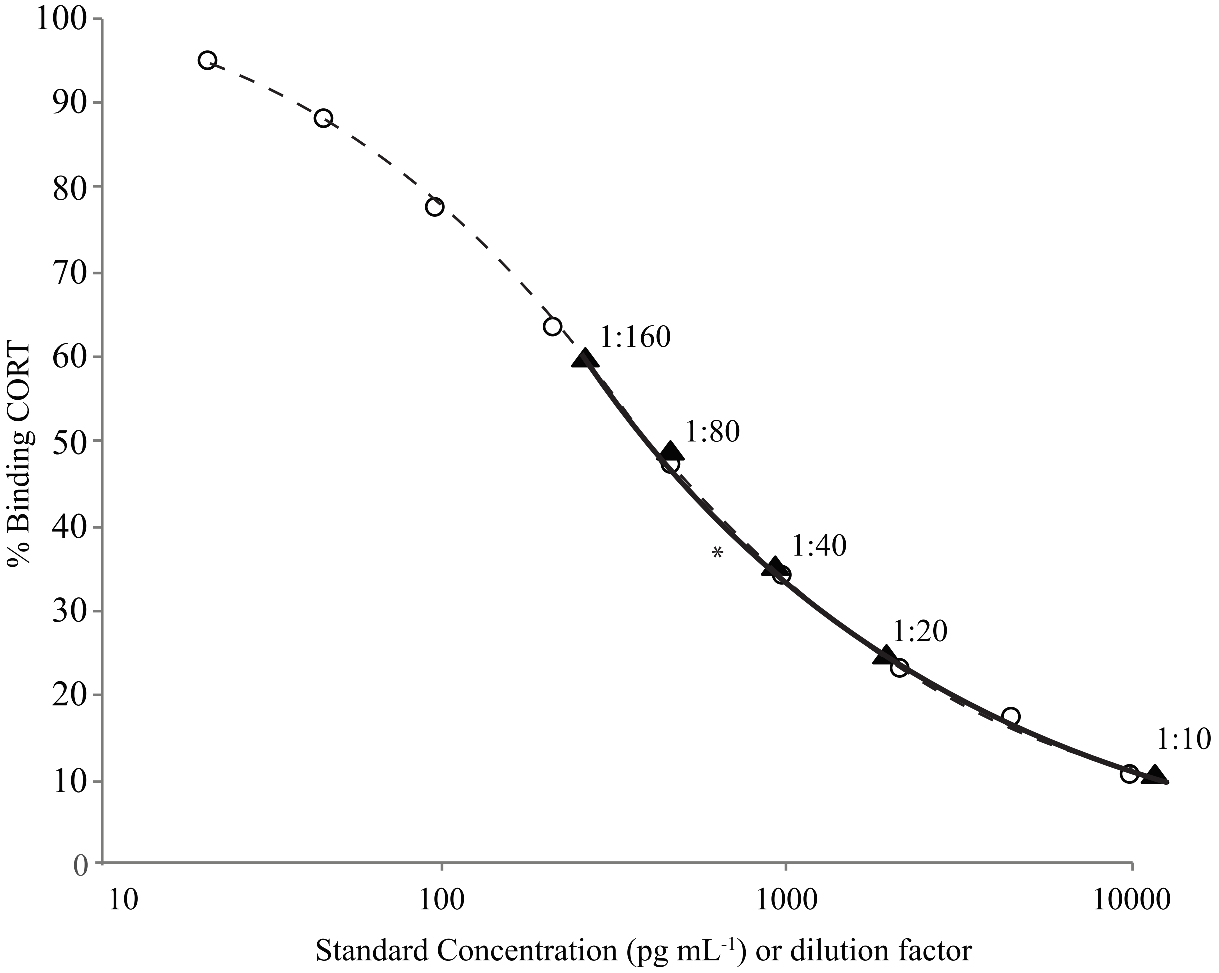

SM3. Parallelism graph for plasma CORT in *S. couchii* using the DetectX® Corticosterone ELISA Kit (Arbor Assays, Ann Arbor, MI; Cat. No. k014). The open circles and solid triangles depict the nine kit standards and the five plasma dilutions (from a pooled sample). Best fit curves were generated using the 4-parameter curve fitting equation in Softmax Pro (Molecular Devices). The optimal (*) dilution factor for plasma was interpolated to be 1:50. A difference of slopes t-test indicated that the slopes of the two curves (with overlapping x-intercepts) were not significantly different (Δ slope = 2.01, SE = 5.3, $t_7 = 0.37$, $p = 0.71$).

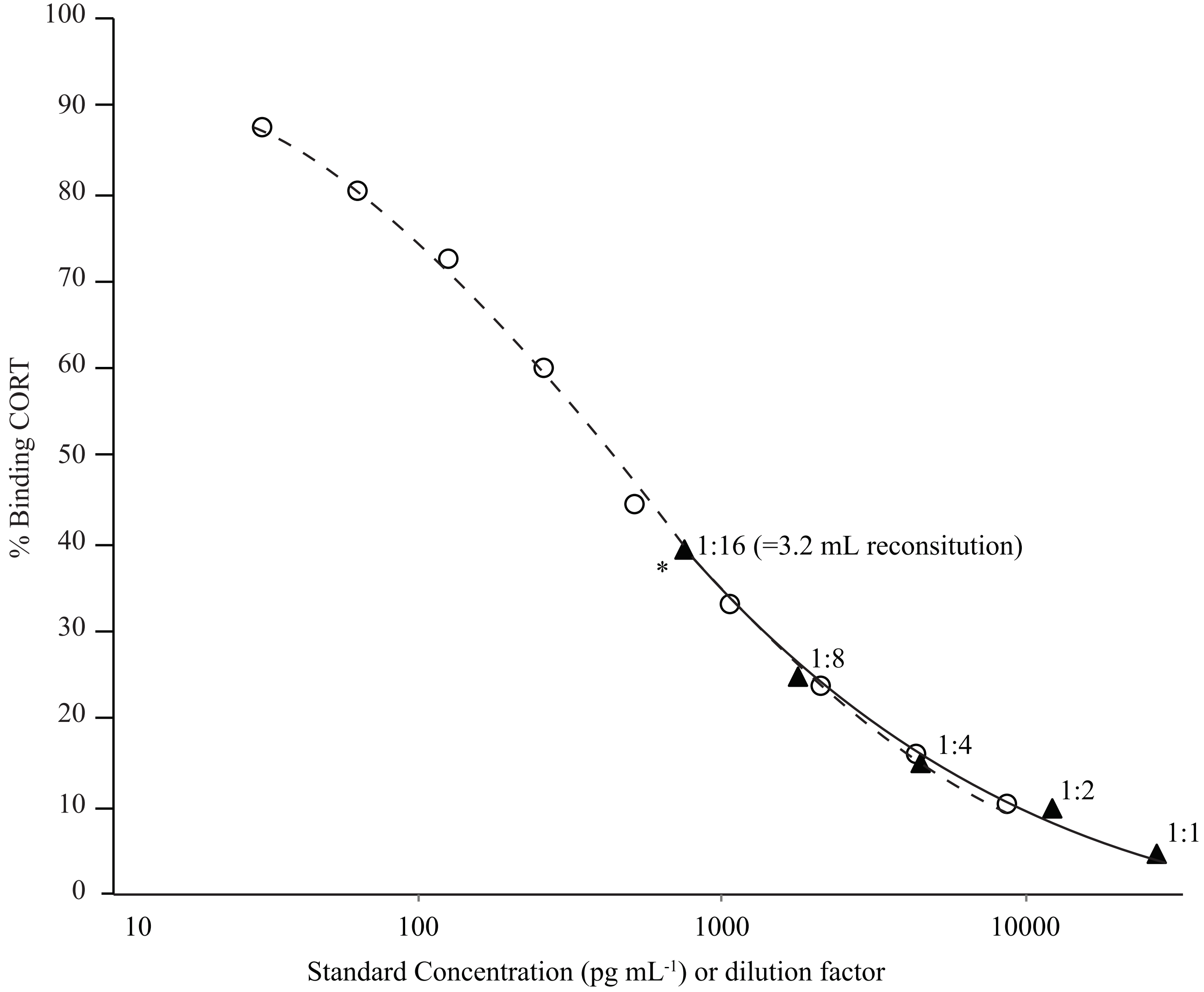

SM4. Parallelism graph for 60 min water baths for water-borne CORT in *S. couchii*using the DetectX® Corticosterone ELISA Kit (Arbor Assays, Ann Arbor, MI; Cat. No. k014). The open circles and solid triangles depict the nine kit standards and the five wather bath reconstitutions (from a pooled sample). Best fit curves were generated using the 4-parameter curve fitting equation in Softmax Pro (Molecular Devices). The optimal (*) reconstitution volume for an average 60 min water baths was 3.2 mL (=1:16 with 1:1 = 200 uL). A difference of slopes t-test indicated that the slopes of the two curves (with overlapping x-intercepts) were not significantly different (Δ slope = 3.28, SE = 6.6, t_5 = 0.49, p = 0.64).

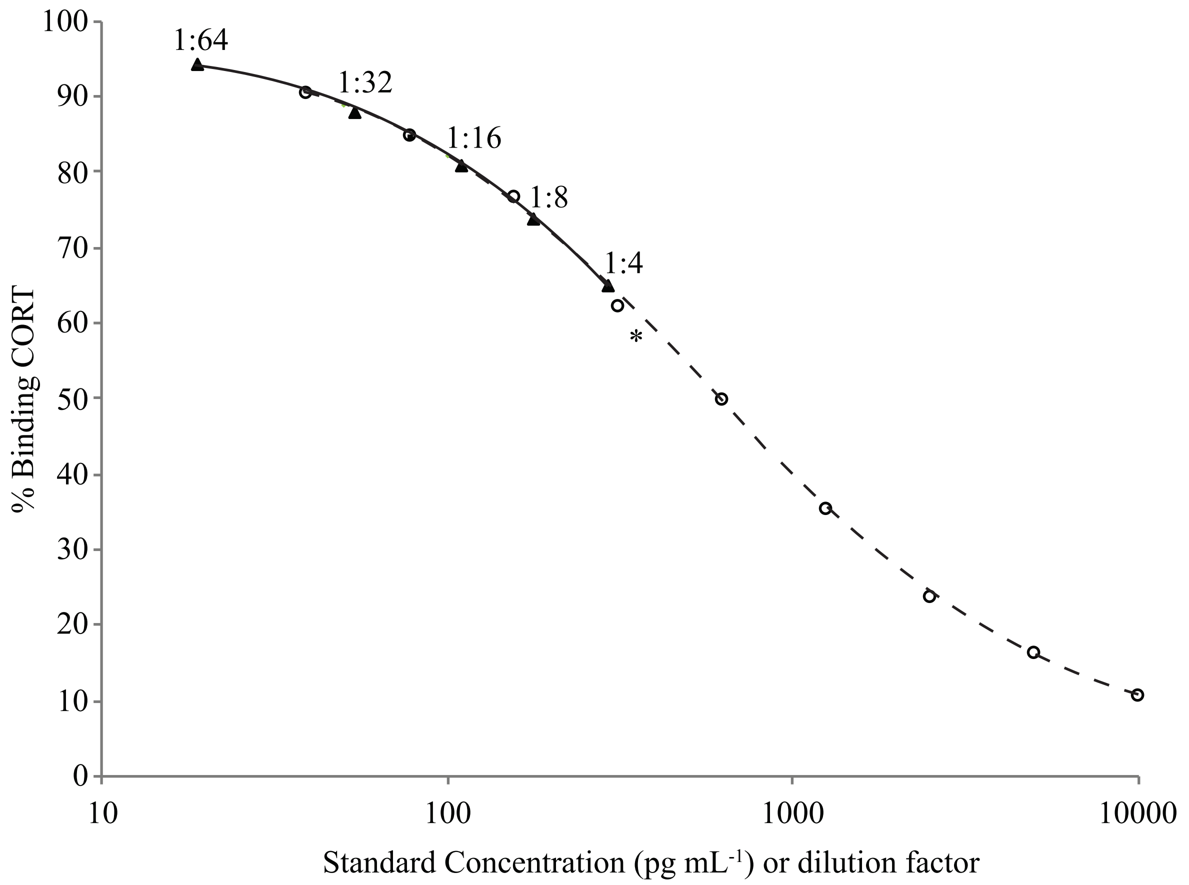

SM5. Parallelism graph for salivary CORT in *S. couchii* using the DetectX® Corticosterone ELISA Kit (Arbor Assays, Ann Arbor, MI; Cat. No. k014). The open circles and solid trianges depict the nine kit standards and the five saliva dilutions (from a pooled sample). Best fit curves were generated using hte 4-parameter curve fitting equation in Softmax Pro (Molecular Devices). The optimal (*) dilution factor for saliva was interpolated to be 1:3. A difference of slopes t-test indicated that the slopes of the two curves (with overlapping x-intercepts) were not significantly different (Δ slope = 0.01, SE = 0.009, t_4 = 1.07, p = 0.34).

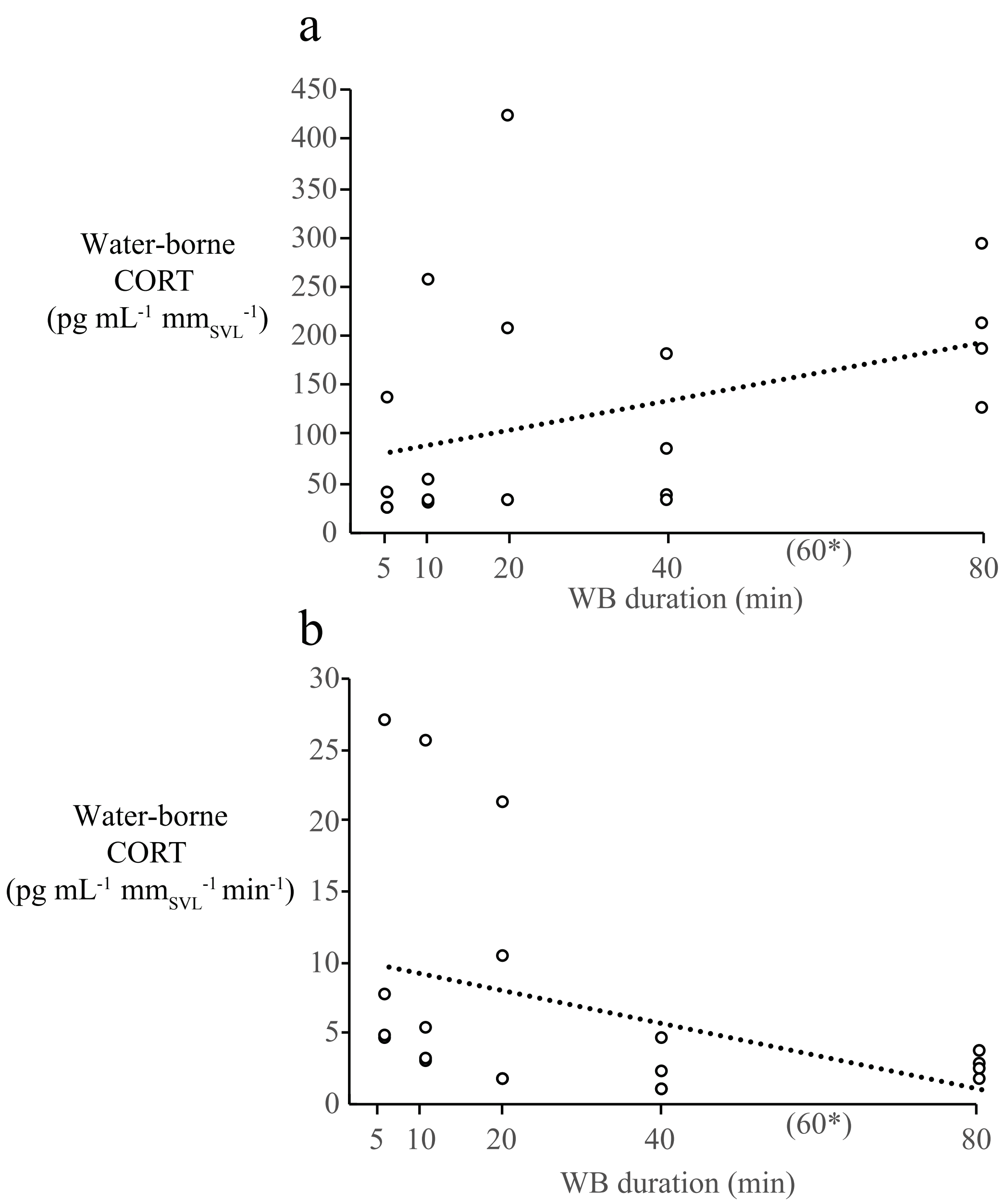

SM6. Water-borne CORT concentrations from individual *S. couchii* (n=20; 10m,10f) sampled for variable duration water baths (n=2m,2f per water bath duration). In (a) the measured concentrations are depicted and the best fit trendline shows that higher levels of CORT are observed with longer water baths. Detectable levels of CORT are present beginning at the shortest duration bath (5 min). The graph in (b) corrects for the the water bath duration in the ordinate by dividing the measured concentration by the duration, thereby illustrating the rate of CORT release. The release rate is least variable at 80 min. To optimize the tradeoff of duration and release rate variability, we elected to conduct all experimental water baths for a 60 min duration (*).

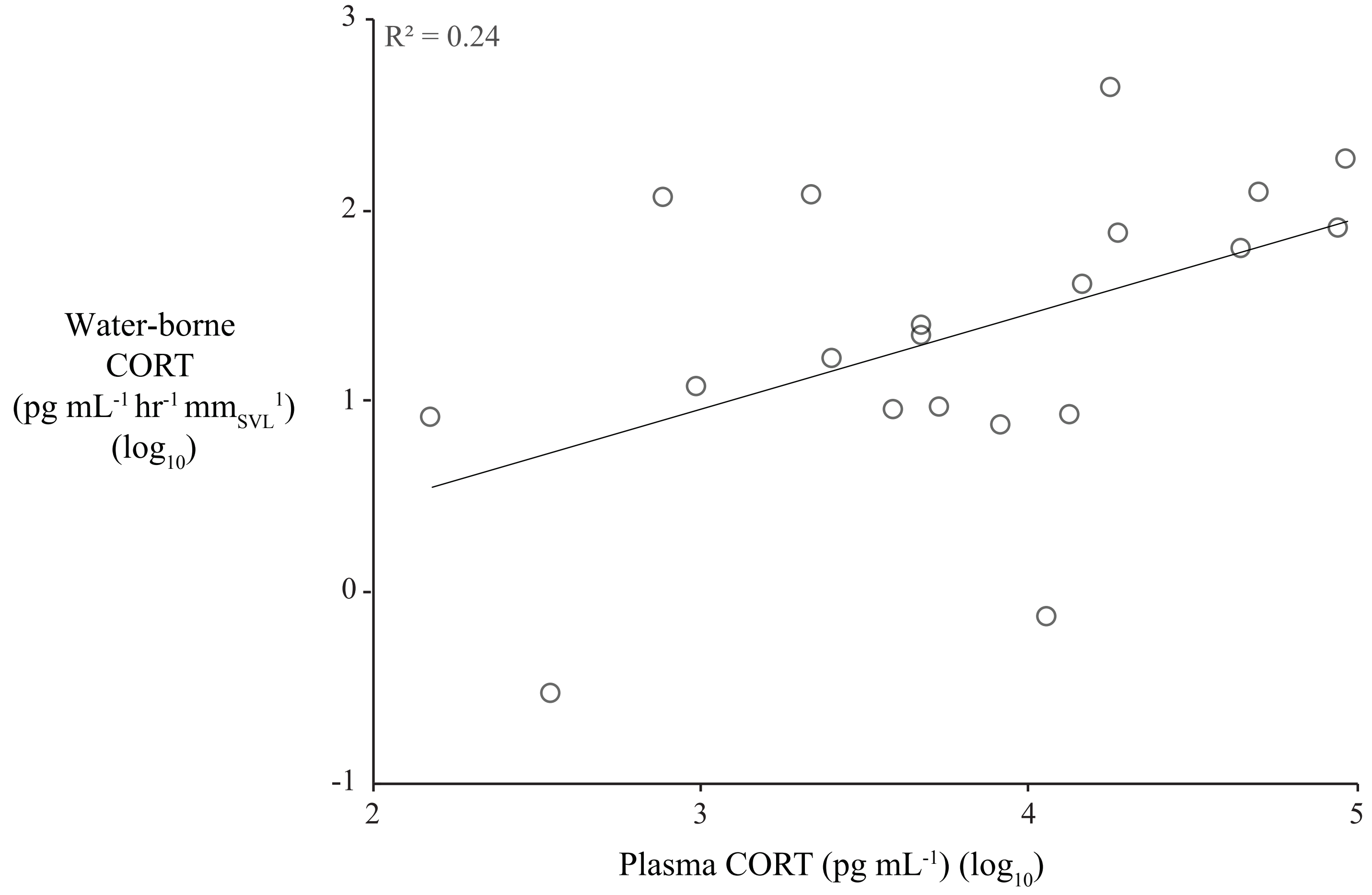

SM7. The linear correlation between plasma and water-borne CORT (n=20; $\log_{10}$-transformed) in unmanipulated frogs. Blood was sampled immediately following the 60 min water bath.

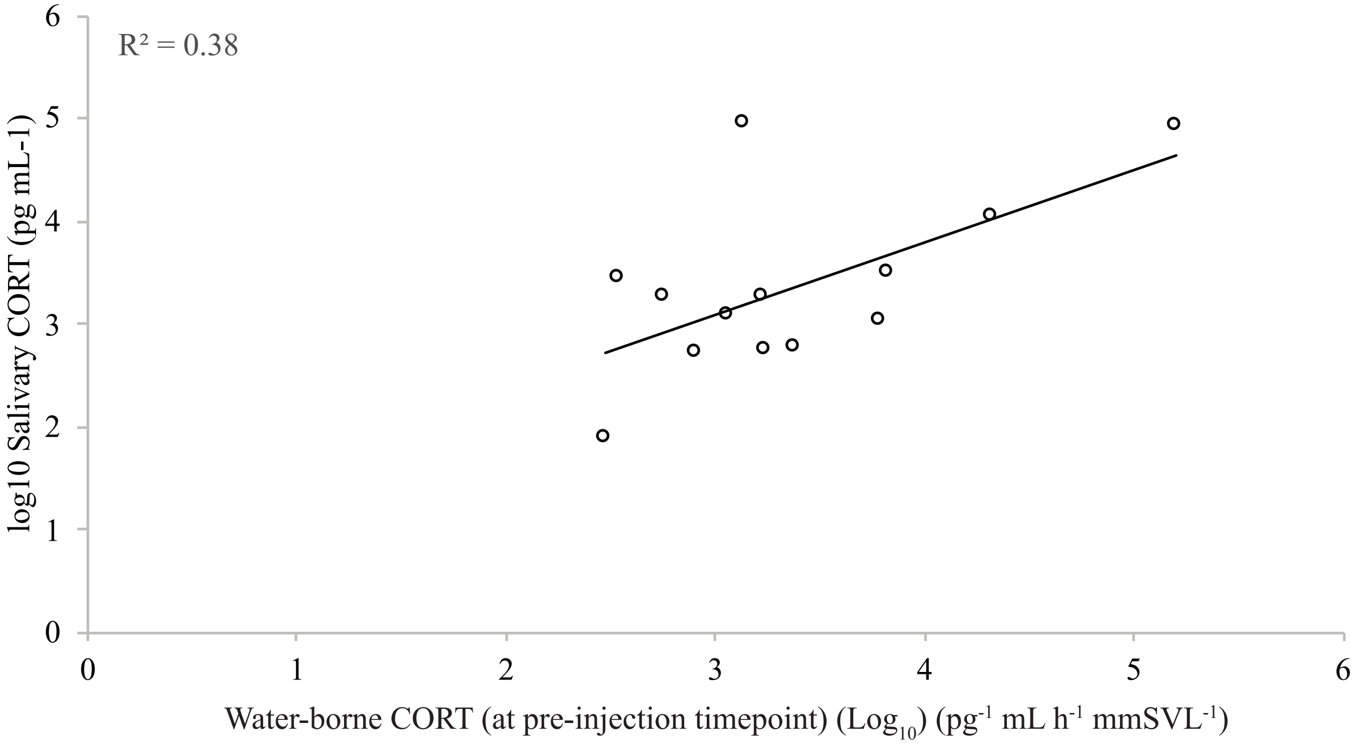

SM8. Water-borne CORT in *S. couchii* was positively correlated with salivary CORT when each sample matrix is collected in close temporal proximity ($R^2 = 0.38$; $F_{1,11}=6.74$, $p = 0.025$).

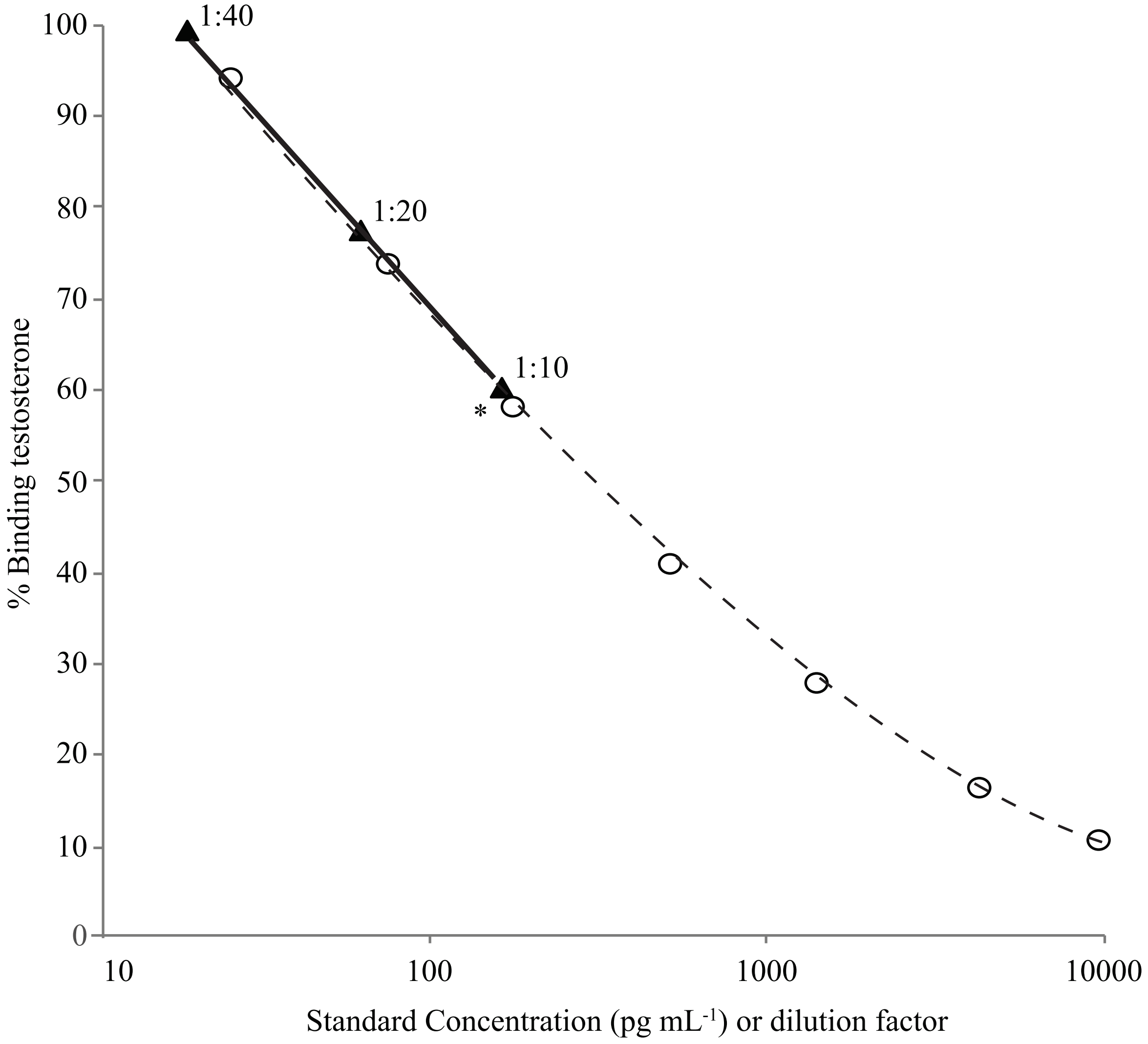

SM9. Parallelism graph for plasma testosterone in *S. couchii* using the DetectX® Testosterone ELISA Kit (Arbor Assays, Ann Arbor, MI; Cat. No. k032). The open circles and solid triangles depict the seven kit standards and the three plasma dilutions (from a pooled sample). Best fit curves were generated using the 4-parameter curve fitting equation in Softmax Pro (Molecular Devices). The optimal (*) dilution factor for plasma was 1:10. A difference of slopes t-test indicated that the slopes of the two curves (with overlapping x-intercepts) were not significantly different (Δ slope = 0.38, SE = 0.44, t_2 = 0.85, p = 0.48).

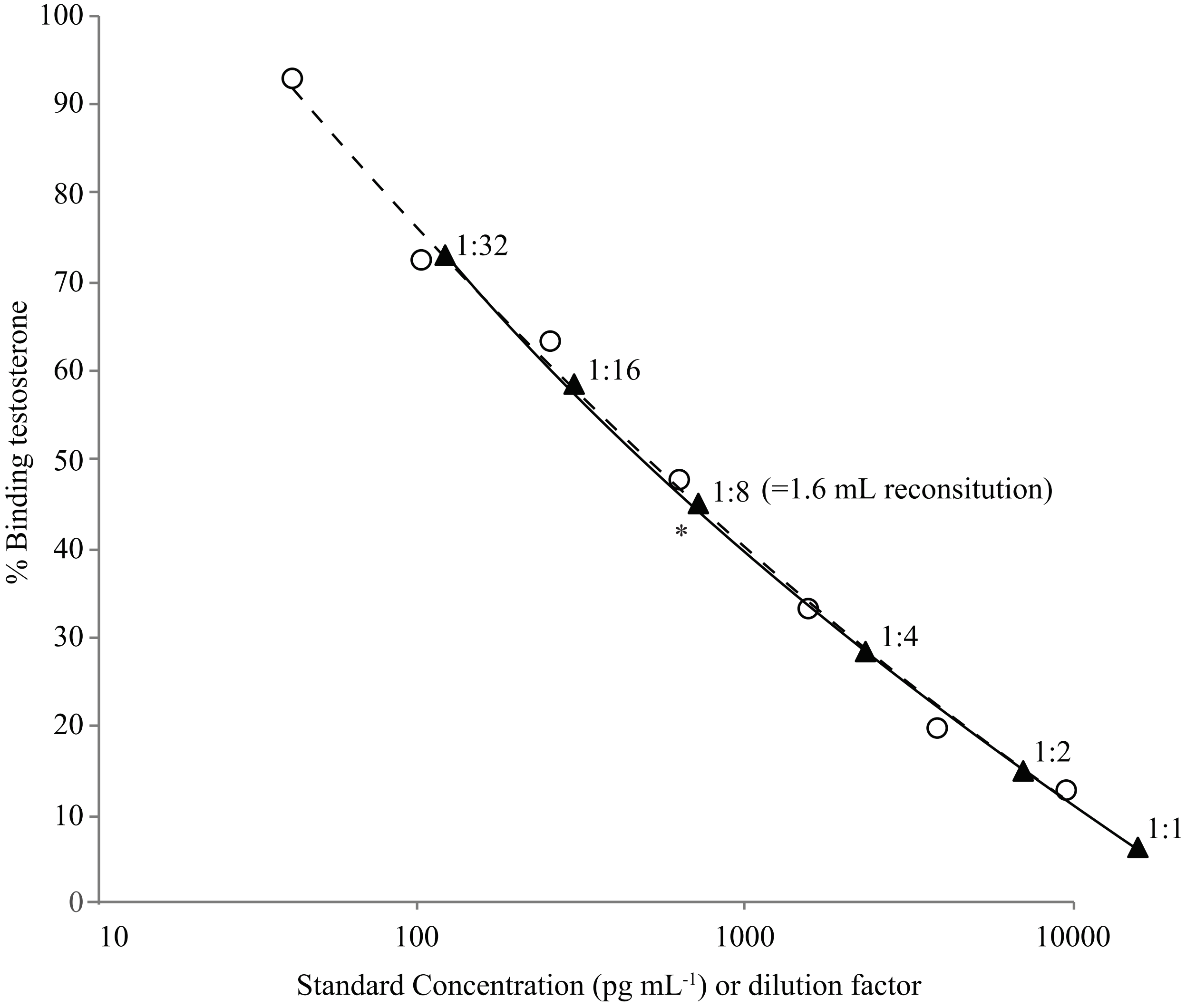

SM10. Parallelism graph for water-borne testosterone in male *S. couchii* using the DetectX® Testosterone ELISA Kit (Arbor Assays, Ann Arbor, MI; Cat. No. k032). The open circles and solid triangles depict the seven kit standards and the six water sample dilutions. Best fit curves were generated using the 4-parameter curve fitting equation in Softmax Pro (Molecular Devices). The optimal (*) dilution factor for a 60 min male water sample was 1:8 (1.6 mL reconstitutions buffer). A difference of slopes t-test indicated that the slopes of the two curves (with overlapping x-intercepts) were not significantly different (Δ slope = 0.56, SE = 2.00, t_8 = 0.28, p = 0.78).

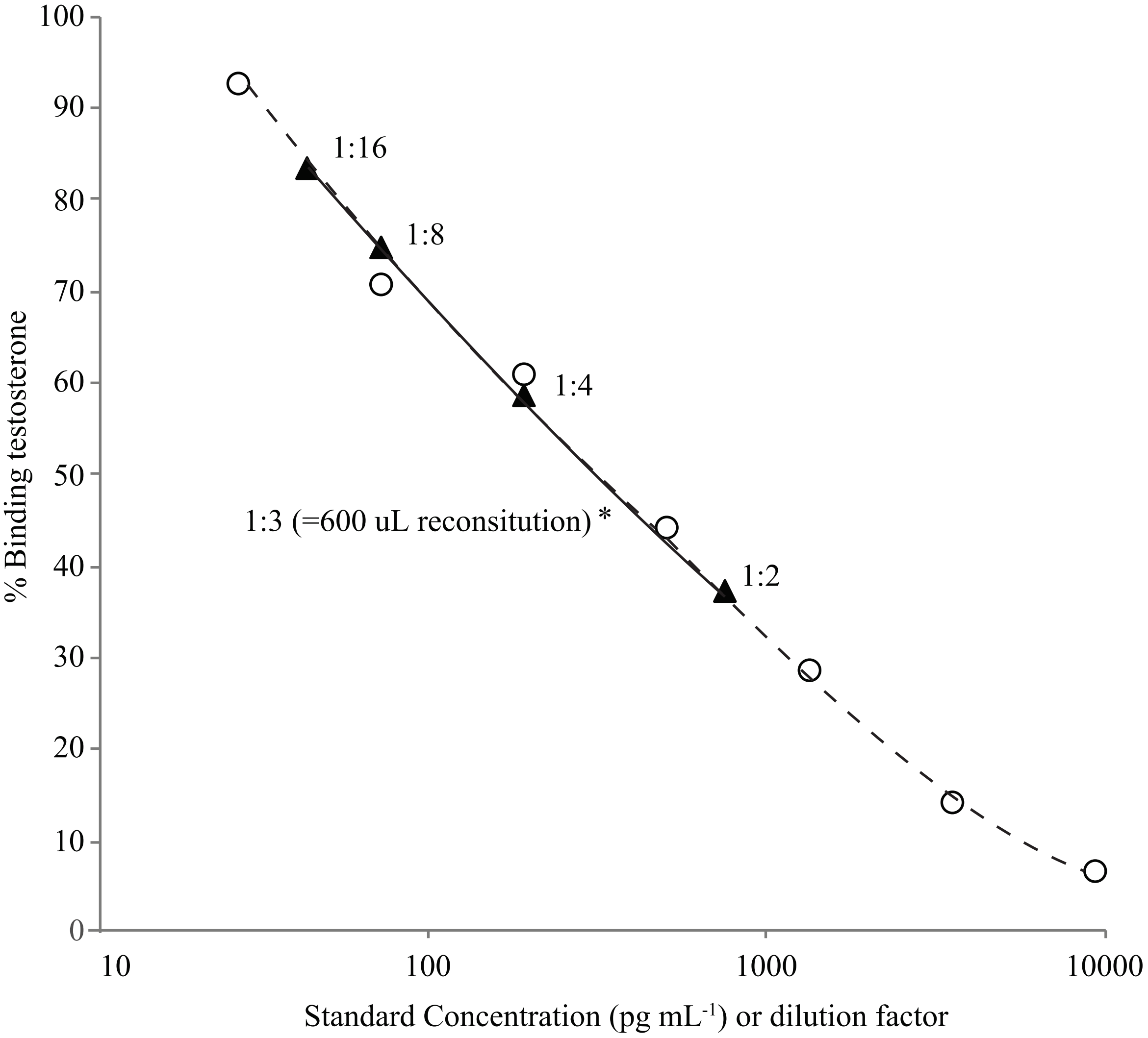

SM11. Parallelism graph for water-borne testosterone in female *S. couchii* using the DetectX® Testosterone ELISA Kit (Arbor Assays, Ann Arbor, MI; Cat. No. k032). The open circles and solid triangles depict the seven kit standards and the five water sample dilutions. Best fit curves were generated using the 4-parameter curve fitting equation in Softmax Pro (Molecular Devices). The optimal (*) dilution factor for a 60 min female water sample was 1:3 (600 uL reconstitutions buffer). A difference of slopes t-test indicated that the slopes of the two curves (with overlapping x-intercepts) were not significantly different (Δ slope = 0.71, SE = 1.06, t_4 = 0.67, p = 0.54).

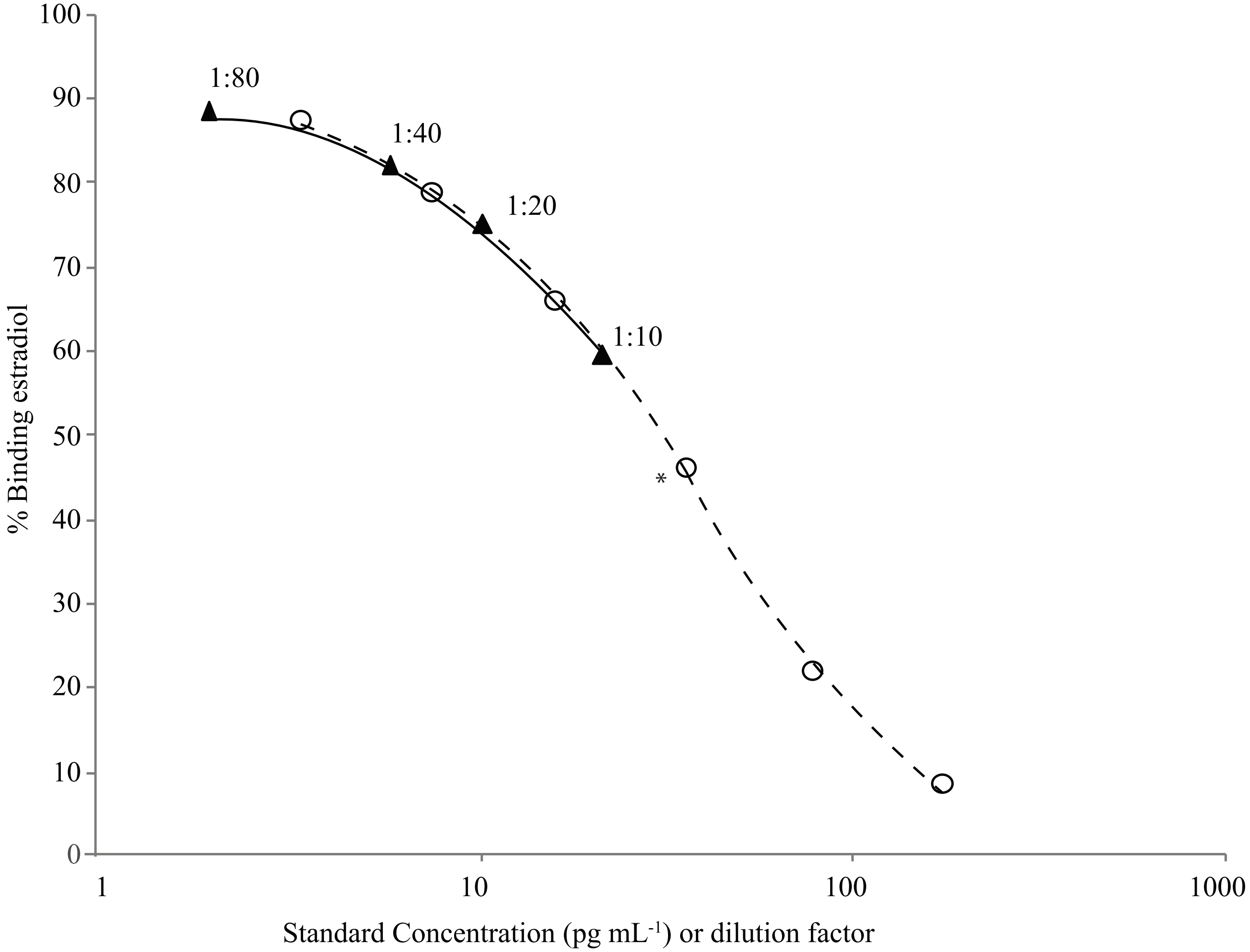

SM12. Parallelism graph for plasma estradiol in *S. couchii* using the DetectX® 17B-estradial ELISA Kit (Arbor Assays, Ann Arbor, MI; Cat. No. kb30). The open circles and solid triangles depict the six kit standards and the four pooled plasma dilutions. Best fit curves were generated using the 4-parameter curve fitting equation in Softmax Pro (Molecular Devices). The optimal (*) dilution factor for plasma was 1:5.
A difference of slopes t-test indicated that the slopes of the two curves (with overlapping x-intercepts) were not significantly different (Δ slope = 5.2, SE = 7.3, $t_3 = 0.71$, $p = 0.53$).

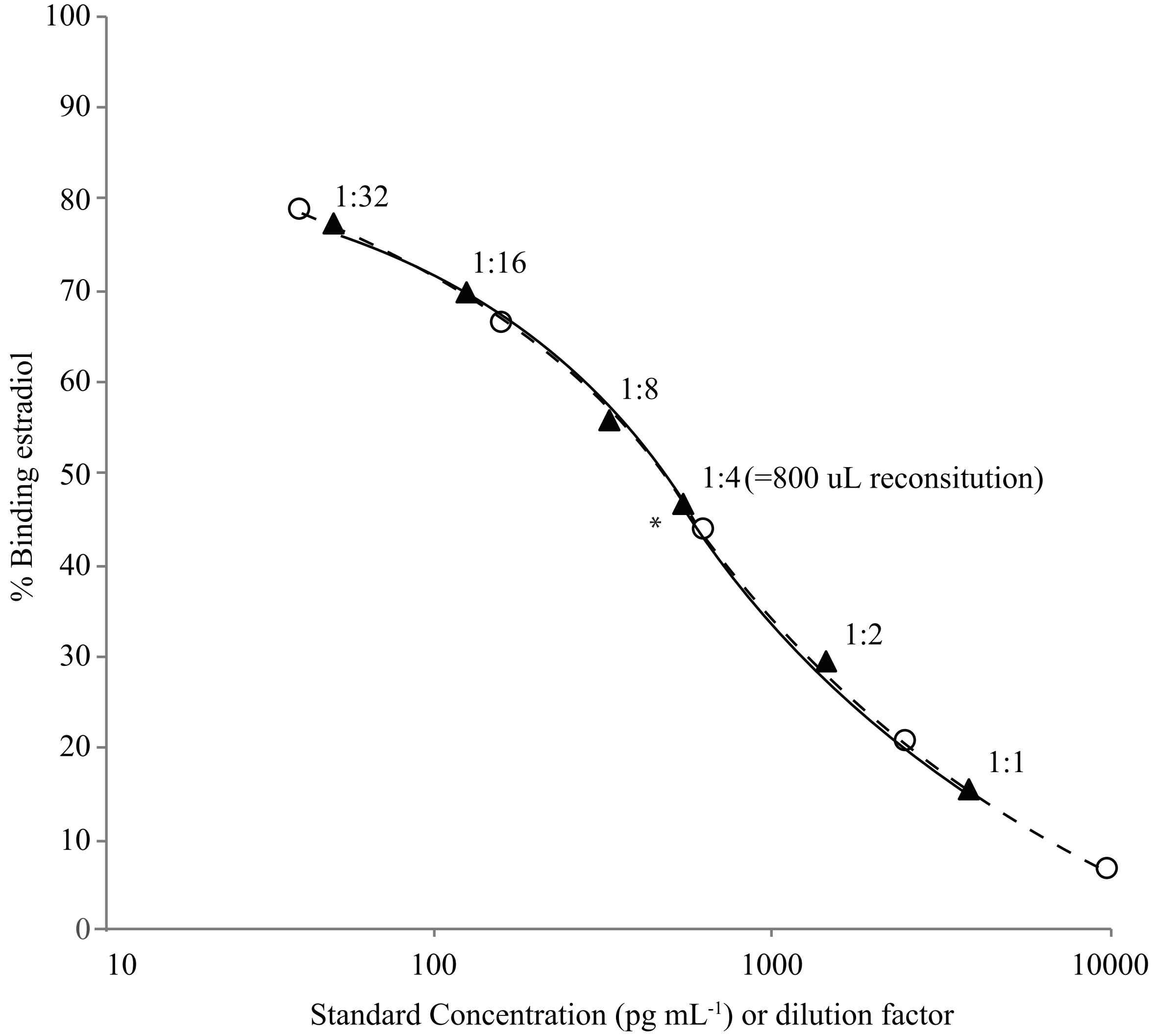

SM13. Parallelism graph for water-borne estradiol in male *S. couchii* using the DetectX® Estradiol ELISA Kit (Arbor Assays, Ann Arbor, MI; Cat. No. k030). The open circles and solid triangles depict the five kit standards and the six water sample dilutions. Best fit curves were generated using the 4-parameter curve fitting equation in Softmax Pro (Molecular Devices). The optimal (*) dilution factor for a 60 min male water sample was 1:4 (800 uL reconstitutions buffer). A difference of slopes t-test indicated that the slopes of the two curves (with overlapping x-intercepts) were not significantly different (Δ slope = 1.2, SE = 3.4, $t_6 = 0.35$, $p = 0.74$).

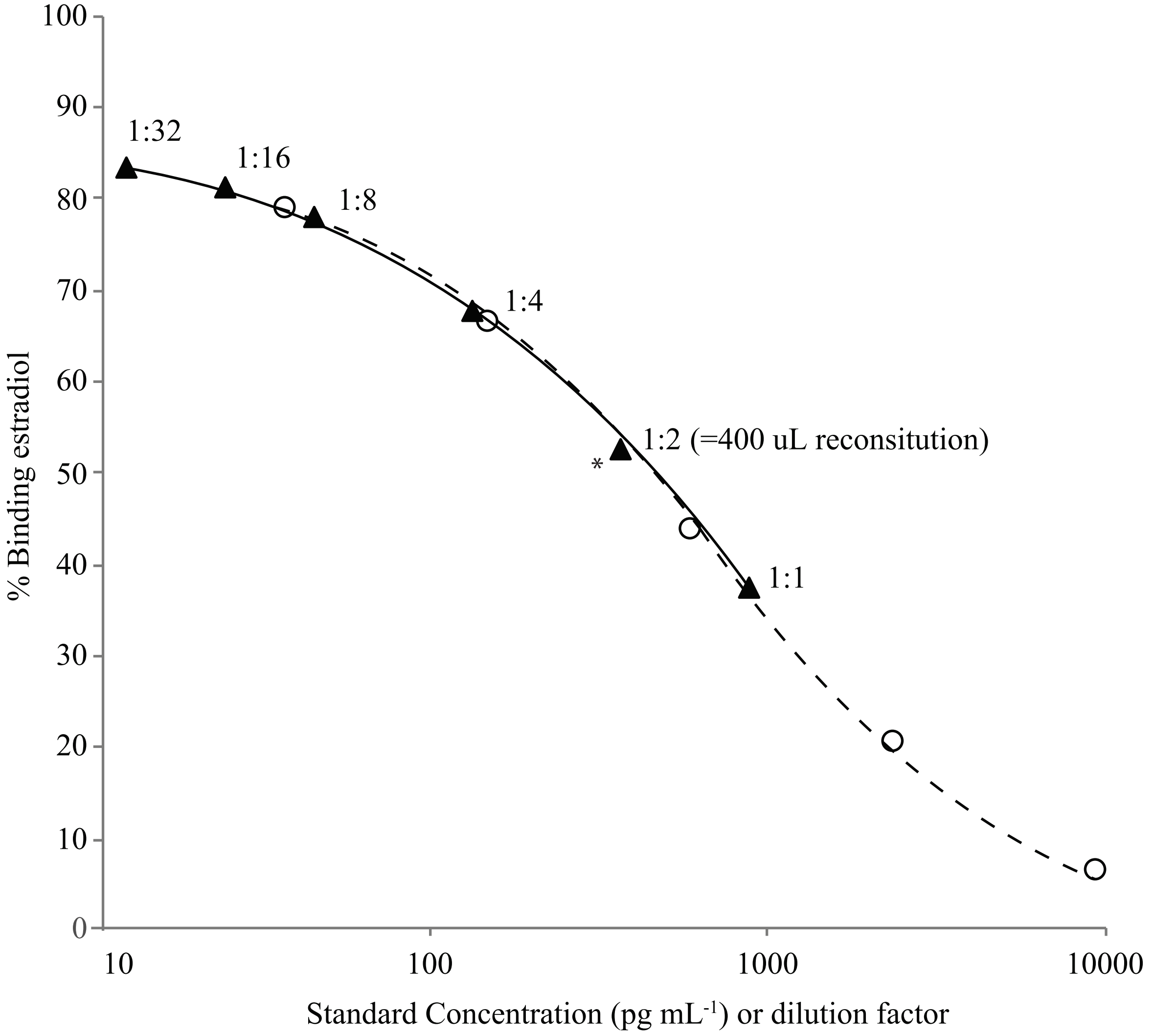

SM14. Parallelism graph for water-borne estradiol in female *S. couchii* using the DetectX® Estradiol ELISA Kit (Arbor Assays, Ann Arbor, MI; Cat. No. k030). The open circles and solid triangles depict the five kit standards and the six water sample dilutions. Best fit curves were generated using the 4-parameter curve fitting equation in Softmax Pro (Molecular Devices). The optimal (*) dilution factor for a 60 min female water sample was 1:2 (400 uL reconstitutions buffer). A difference of slopes t-test indicated that the slopes of the two curves (with overlapping x-intercepts) were not significantly different (Δ slope = 4.3, SE = 5.7, $t_5 = 0.75$, $p = 0.49$).

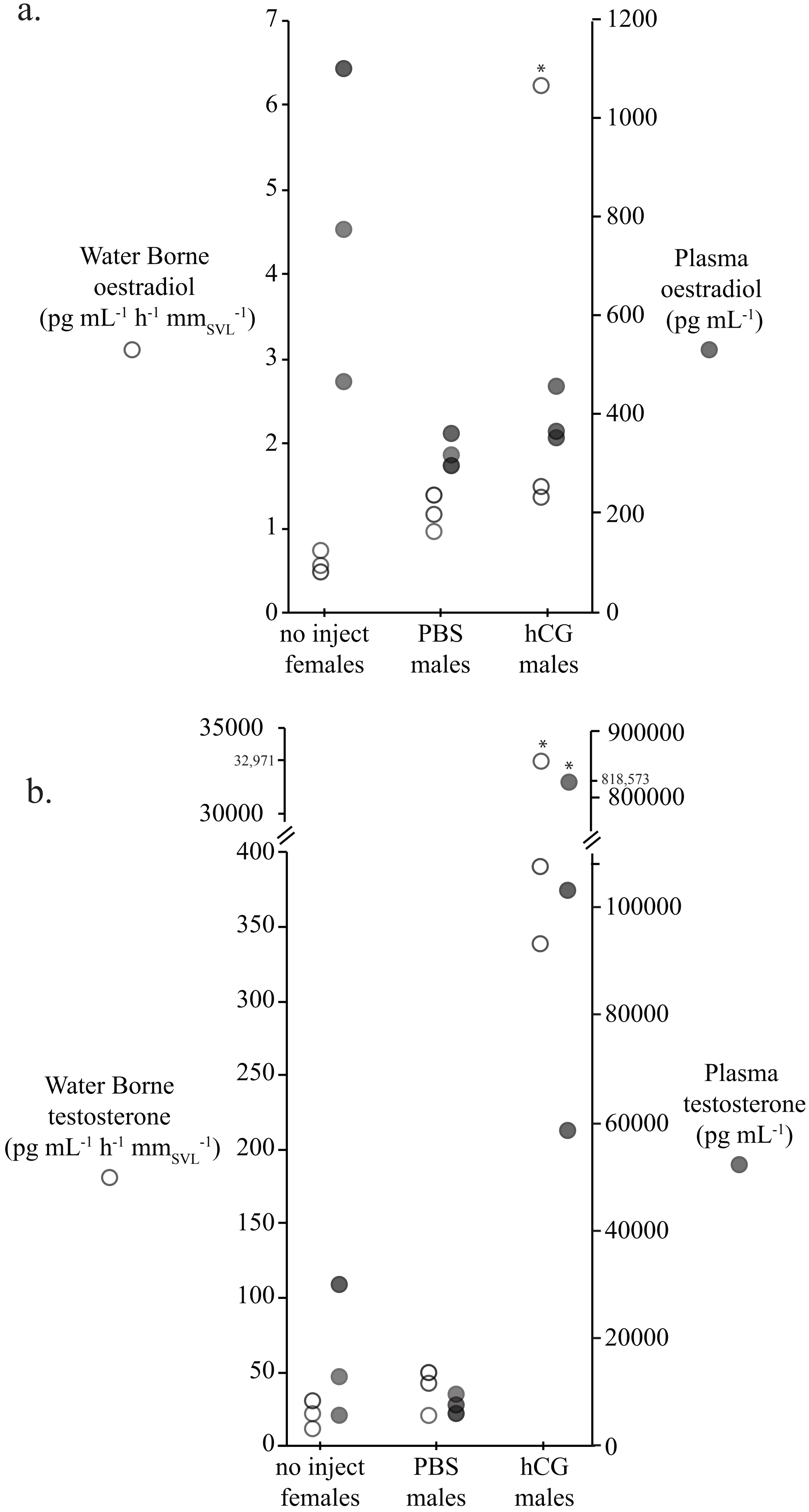

SM15. Estradiol (a) and testosterone (b) levels from water samples (left y-axis and open circles) and plasma samples (right y-axis and closed circles) from frogs from each of the three treatments in the hCG challenge experiment. The water sample from the hCG male with the highest estradiol (*) was the same sample with very high water-borne and plasma testosterone (*) in (b). Note the interupted y-axes for water-borne and plasma testosterone in (b).

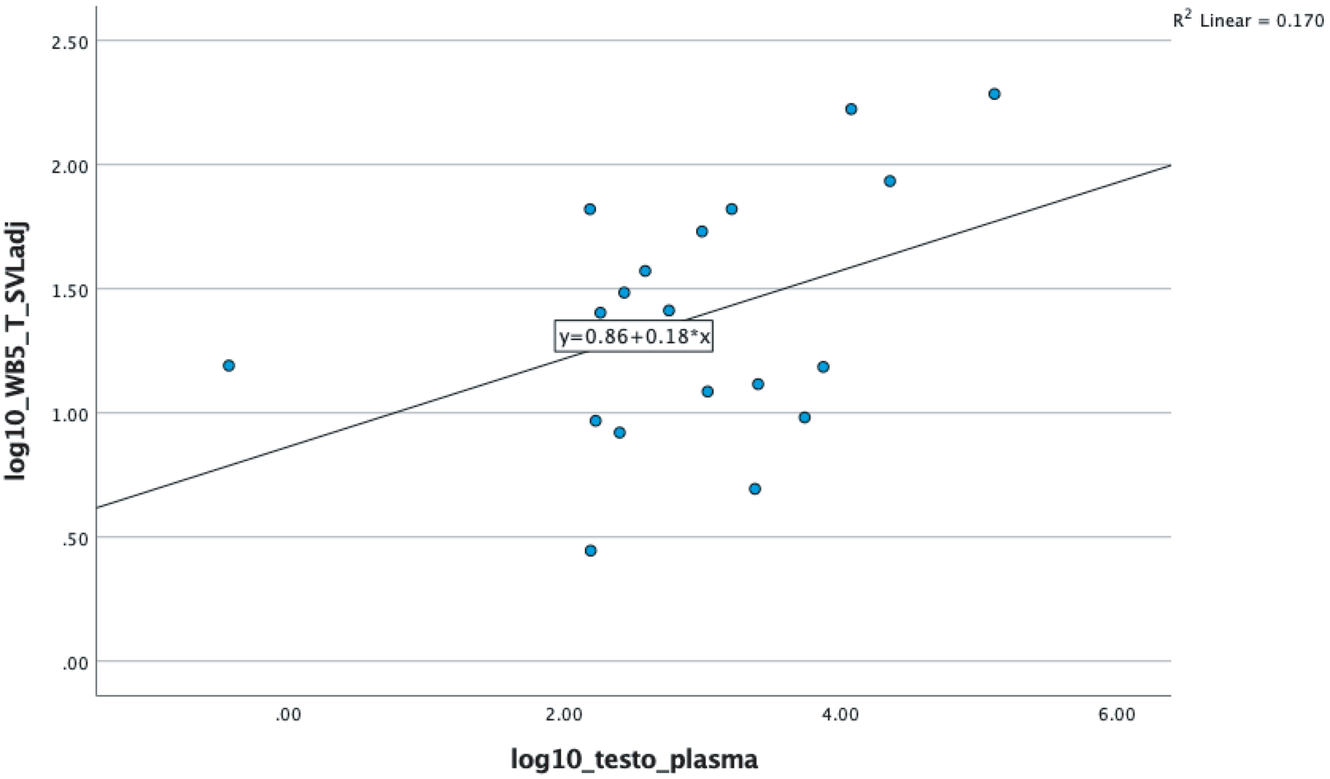

SM16. Linear correlations of plasma oncentrations of testosterone and water-borne testosterone at water bath #5 time-point in the GnRH experiment (R^2=0.17; all p=0.07; n=20); both treatments and sexes included).

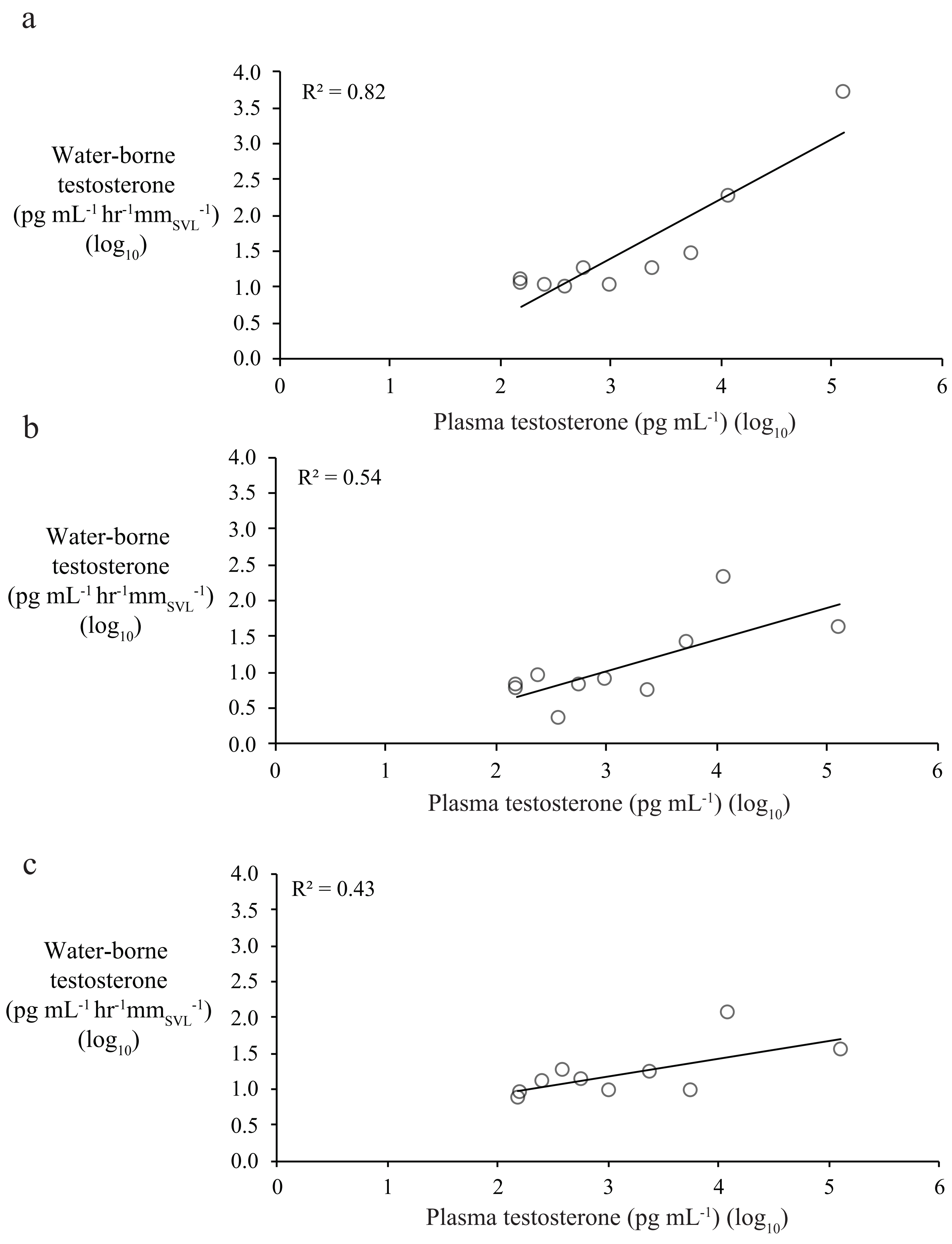

SM17. Circulating concentrations of testosterone were significantly correlated with water-borne testosterone in male frogs from the GnRH Challenge experiment at the second (a), fourth (b) and sixth (c) water bath time-points.

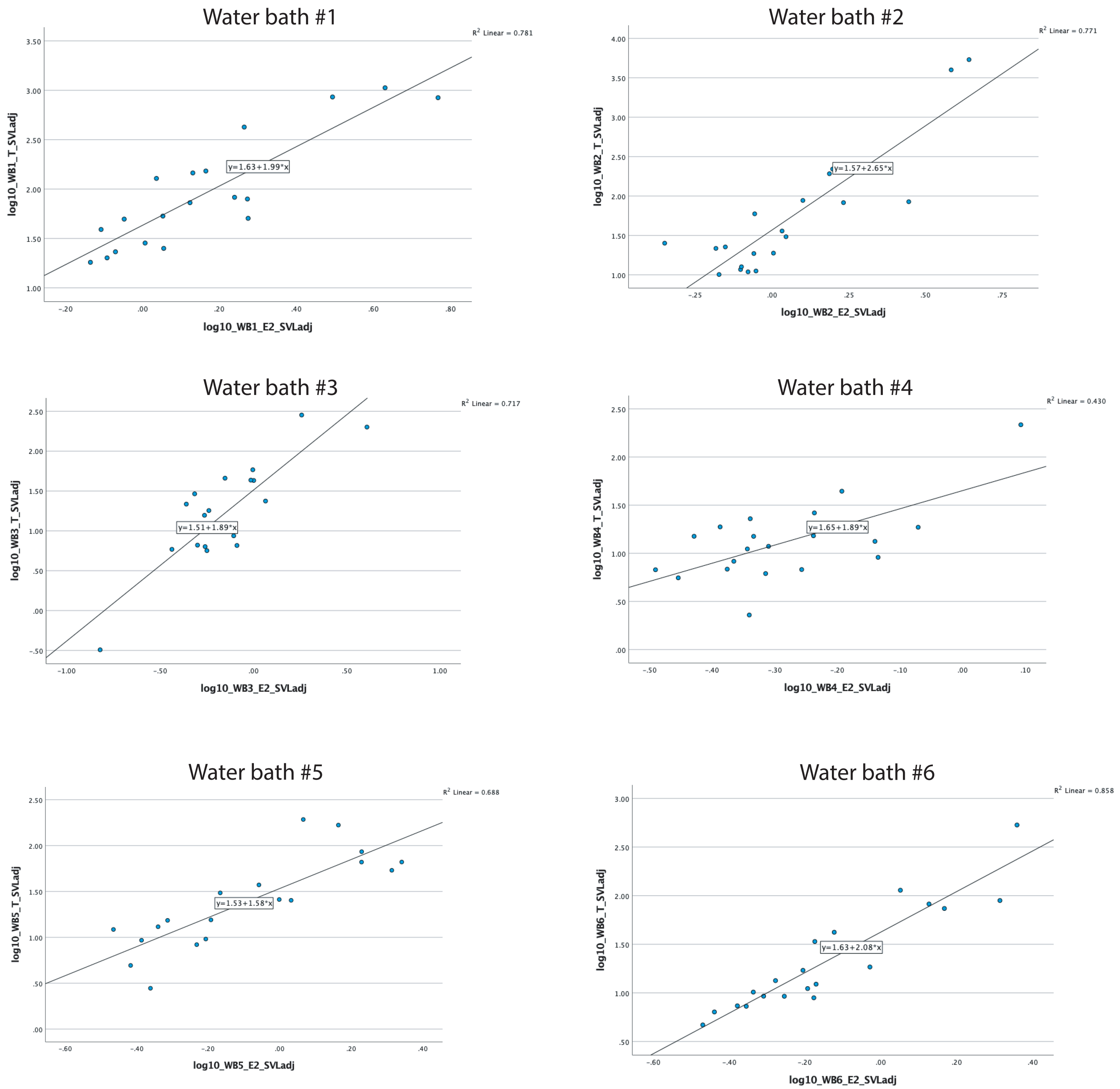

SM18. Linear correlations of water borne concentrations of estradiol and testosterone for each of the six repeated water bath timepoints in the GnRH Challenge Experiment (R^2=0.43 - 0.85; all p<0.002; n=20); both treatments and sexes included).
